# Calcium-induced conformational changes of the regulatory domain of human mitochondrial aspartate/glutamate carriers

**DOI:** 10.1038/ncomms6491

**Published:** 2014-11-20

**Authors:** Chancievan Thangaratnarajah, Jonathan J. Ruprecht, Edmund R. S. Kunji

**Affiliations:** 1 The Medical Research Council, Mitochondrial Biology Unit, Wellcome Trust/MRC Building, Hills Road, Cambridge CB2 0XY, UK

## Abstract

The transport activity of human mitochondrial aspartate/glutamate carriers is central to the malate–aspartate shuttle, urea cycle, gluconeogenesis and myelin synthesis. They have a unique three-domain structure, comprising a calcium-regulated N-terminal domain with eight EF-hands, a mitochondrial carrier domain, and a C-terminal domain. Here we present the calcium-bound and calcium-free structures of the N- and C-terminal domains, elucidating the mechanism of calcium regulation. Unexpectedly, EF-hands 4–8 are involved in dimerization of the carrier and form a static unit, whereas EF-hands 1–3 form a calcium-responsive mobile unit. On calcium binding, an amphipathic helix of the C-terminal domain binds to the N-terminal domain, opening a vestibule. In the absence of calcium, the mobile unit closes the vestibule. Opening and closing of the vestibule might regulate access of substrates to the carrier domain, which is involved in their transport. These structures provide a framework for understanding cases of the mitochondrial disease citrin deficiency.

## Abstract (web summary)

Human mitochondrial aspartate/glutamate carriers, citrin and aralar, are regulated by calcium. Here, the authors report the dimeric structure of calcium-free and -bound versions of the regulatory domains to elucidate calcium-dependent conformational changes that could regulate access of substrate to the carrier domain.

## Graphical abstract

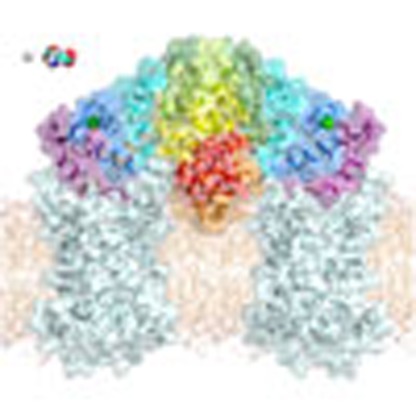

The mitochondrial aspartate/glutamate carriers import glutamate together with a proton into the mitochondrial matrix and export aspartate^1^,^2^,^3^,^4^,^5^,^6^. The transport step was discovered more than 45 years ago^7^, but it was another 30 years before the molecular identity was established^8^,^9^,^10^. The transport protein is a member of the mitochondrial carrier family and plays a central role in several important ubiquitous and tissue-specific processes. In humans there are two isoforms, which are expressed in a tissue-specific pattern; aralar (SLC25A12 or AGC1) is predominately expressed in excitable tissues^8^ and citrin (SLC25A13 or AGC2) in non-excitable tissues^9^,^10^. The aspartate/glutamate carriers play a key role in the operation of the malate–aspartate shuttle, which is required for the import of reducing equivalents into the mitochondrion^1^,^11^,^12^. In addition, they are crucial for the supply of mitochondrial aspartate to the cytosol. In liver, cytosolic aspartate is required for the urea cycle^11^, which is crucial for the production of urea from the deamination of amino acids. Cytosolic aspartate is also required for the conversion of oxoglutarate to oxaloacetate, a crucial step in gluconeogenesis from lactate and alanine^13^,^14^. In neurons, aralar catalyses the efflux of aspartate required for the production of *N*-acetyl-aspartate for the synthesis of myelin^15^. Mutations in the *SLC25A12* and *SLC25A13* genes cause global cerebral hypomyelination (OMIM: 603667)^16^ and citrin deficiency (OMIM: 603859)^9^, affecting myelin synthesis and the urea cycle, respectively.

Following the identification of the genes, analysis of the molecular features showed that the transporters are calcium-regulated^6^. Unlike other members of the mitochondrial carrier family, the aspartate/glutamate carriers have a three-domain structure, consisting of a hydrophilic N-terminal domain with EF-hand motifs, a mitochondrial carrier domain^8^,^9^,^10^ and a C-terminal domain of unknown function^8^. The N-terminal domain has been proposed to contain eight EF-hand motifs^17^ that have sequence identities of 25–30% to other calcium-binding proteins, and it has been demonstrated to bind calcium by ^45^Ca^2+^-overlay experiments^8^,^10^. Proteolysis experiments with proteinase K in the brain and liver mitoplasts have shown that the N-terminal domain protrudes into the intermembrane space, providing binding sites for extra-mitochondrial calcium^6^. Binding of calcium leads to the stimulation of the transport activity and consequently the malate-aspartate shuttle^6^,^17^,^18^,^19^,^20^. It has been suggested that the N-terminal domains of aralar and citrin are dissimilar, as reflected in differences in the calcium sensitivity of upregulation and in the putative numbers of functional EF-hands^17^. The carrier domain, which accounts solely for the transport activity^6^, shares 20–30% sequence identity with other members of the mitochondrial carrier family^8^,^9^,^10^ and is anticipated to adopt a similar structure to the bovine and yeast ADP/ATP carriers^21^,^22^. The yeast ADP/ATP carrier is structurally monomeric in the membrane and in detergent^22^,^23^,^24^,^25^, and functions as a monomer^25^. The bovine orthologue has also been shown to be structurally monomeric^21^,^26^. On the basis of related protein properties, other mitochondrial carriers are likely to be monomeric in structure and function^27^. The C-terminal domain has been suggested to be an extra transmembrane helix of the carrier domain on the basis of its hydrophobic nature^8^.

Here we present X-ray structures of the regulatory domains of the aspartate/glutamate carriers in calcium-bound and calcium-free conformations. The regulatory domains consist of eight EF-hands in a unique arrangement resembling an arch. Surprisingly, the aspartate/glutamate carriers dimerize via EF-hands 4–8 of the N-terminal domain, which have evolved from their canonical function of calcium binding to form a large and static dimerization interface. Out of the eight EF-hands, only EF-hand 2 is involved in calcium binding in both citrin and aralar. In the calcium-bound state, an amphipathic helix of the C-terminal domain binds to a hydrophobic groove in the N-terminal domain, opening a vestibule for the substrates to enter the carrier domain. In the calcium-free state EF-hands 1–2 close the hydrophobic groove with the loop between EF-hands 2 and 3 being the bending region, sealing the vestibule. On the basis of these observations we propose a mechanism by which calcium binding regulates the transport activity of the aspartate/glutamate carriers.

## Results

### Human mitochondrial aspartate/glutamate carriers are dimeric

**Figure 1:**
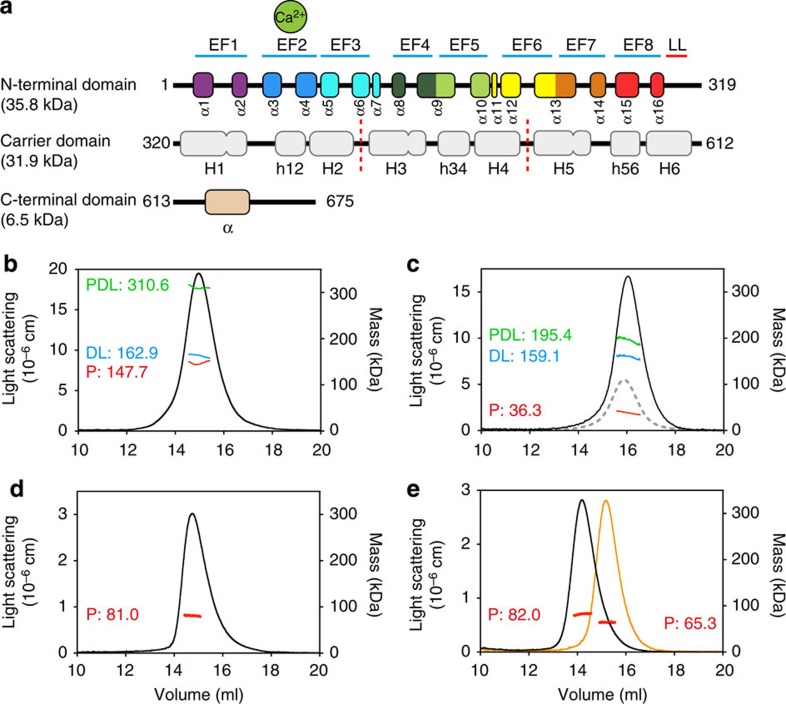
The overall structure and masses of the full-length mitochondrial aspartate/glutamate carrier and its domains. (**a**) The three-domain structure of the aspartate/glutamate carrier isoform 2, citrin, consisting of the N-terminal domain (residues 1–319), carrier domain (residues 320–612) and the C-terminal domain (residues 613–675). The α-helices of EF-hands 1–8 in the N-terminal domain (blue lines) are shown in a rainbow colour scheme. The calcium ion, bound by EF-hand 2, is indicated by a green circle. The α-helices of the transporter domain are shown in light grey, based on secondary-structure predictions and similarity to the structures of the bovine^21^ and yeast ADP/ATP carriers^22^. Red dotted vertical lines highlight the borders of the three homologous repeats. The α-helix of the C-terminal domain is shown in wheat colour. Light scattering traces of (**b**) citrin in lauryl maltose neopentyl glycol, (**c**) carrier domain of citrin (black line) and the yeast ADP/ATP carrier Aac2p in dodecyl-maltoside (grey dashed line), (**d**) N- and C-terminal domain fusion of citrin, and (**e**) N-terminal domain (orange) and N- and C-terminal domain fusion of aralar (black). The masses of the proteins, as determined by SEC-MALLS, are shown in red. The masses of the associated protein-detergent-lipid micelles (PDL) and detergent-lipid micelles (DL) are shown in green and blue, respectively, where relevant.

The two human isoforms citrin (74 kDa) and aralar (75 kDa) are 77% identical (Supplementary Fig. 1), and have the same three-domain structure (Fig. 1a). They consist of an N-terminal domain with eight predicted EF-hand motifs (citrin residues 1–319, 36 kDa), a carrier domain (citrin residues 320–612, 32 kDa) and a C-terminal domain (citrin residues 613–675, 6 kDa). Citrin was purified in lauryl maltose neopentyl glycol^28^ (Supplementary Fig. 2a), and its mass was determined by size-exclusion chromatography coupled to multi-angle laser light scattering (SEC-MALLS)^29^. Surprisingly, the protein was found to have a mass of 147.7±1.7 kDa, twice the theoretical mass of 74 kDa, demonstrating that the full-length carrier was dimeric (Fig. 1b). This result was unexpected, as other mitochondrial carriers are monomeric, based on their sequence properties^27^, structure^21^,^22^,^23^ and functional features^25^,^27^,^30^. To determine which domain was involved in dimerization, each was purified in isolation and assessed by SEC-MALLS (Supplementary Fig. 2b–f). It was not possible to determine accurately the mass of the carrier domain in lauryl maltose neopentyl glycol, as the protein was partially aggregated, but the majority was monomeric (39.9±1.4 kDa). The carrier domain was monodisperse in dodecyl-maltoside and had a mass of 36.3±0.5 kDa (Fig. 1c and Supplementary Fig. 2b), similar to the calculated mass of 32 kDa and to carboxyatractyloside-inhibited Aac2p, which is known to be monomeric^24^ (Fig. 1c and Supplementary Fig. 2c). The results show that the carrier domain in isolation is monomeric. Thus, the dimerization of the full-length carrier is mediated via the N- and/or C-terminal domain. It was not possible to express and purify the N-terminal domain of citrin on its own. The carrier domain has a similar topology to other mitochondrial carriers, consisting of six transmembrane helices (Fig. 1a)^30^. Thus, the N- and C-terminal domains are both in the intermembrane space^31^,^32^, as shown experimentally for the N-terminal domain^6^. The N-terminal domain ends at the first helix of the carrier domain, whereas the C-terminal domain starts on the sixth helix of the carrier (Fig. 1a), which are adjacent structurally, as shown for the mitochondrial ADP/ATP carrier^21^,^22^. A fusion protein was engineered, which mimics this arrangement by linking the two domains via a TEV protease cleavage sequence. The N- and C-terminal domain fusion of citrin and aralar were expressed, purified (Supplementary Fig. 2d,e), and assessed by SEC-MALLS. The masses of the domain fusions of citrin and aralar were 80.9±0.8 kDa and 82.0±0.9 kDa, respectively, twice the theoretical mass, demonstrating that the fusion proteins were dimeric (Fig. 1d,e). The N-terminal domain of aralar on its own could be expressed and purified (Supplementary Fig. 2f), and was shown to be 65.3±0.3 kDa (Fig. 1e), also a dimer. Taken together these results show that the N-terminal domains mediate the dimerization of the full-length aspartate/glutamate carriers.

### Architecture of the N- and C-terminal domain fusion of citrin

**Figure 2:**
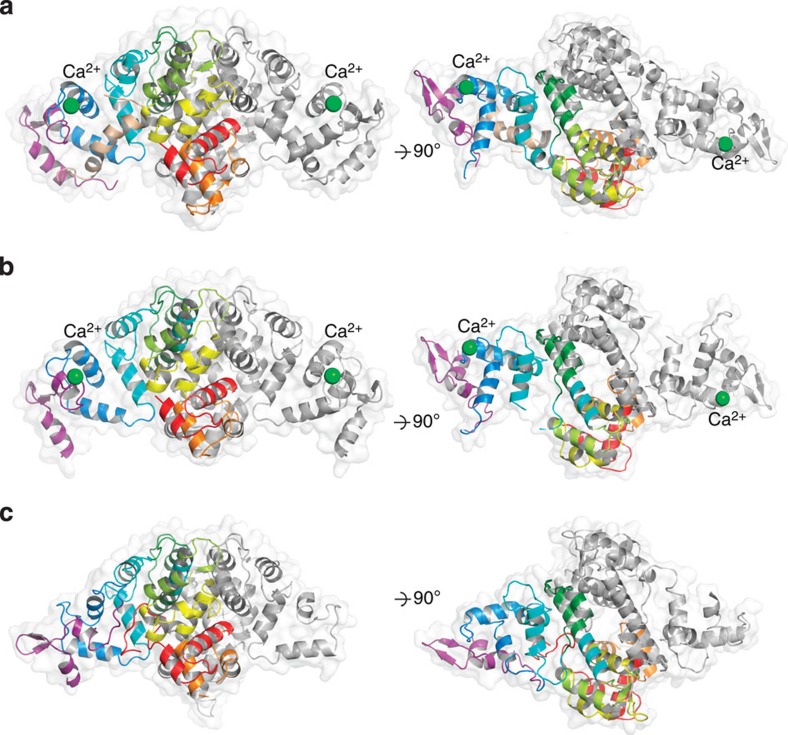
Architecture of the regulatory domain of citrin and aralar. (**a**) calcium-bound N- and C-terminal domain of citrin, and (**b**) calcium-bound and (**c**) calcium-free state of the N-terminal domain of aralar. Two views of the domains are shown in cartoon representation, related by a 90-degree rotation. Protomer A is coloured according to the scheme in Fig. 1a, whereas protomer B is coloured in light grey. Bound calcium ions are shown as green spheres. Protomers A and B of the calcium-free state structure shown here correspond to chains B and A, respectively.

To determine the molecular basis for dimerization and calcium regulation, we crystallized the N- and C-terminal domain fusion of citrin and solved the structure to 2.4 Å resolution, with phases determined by multiple isomorphous replacement with anomalous scattering (MIRAS). The statistics are shown in Supplementary Table 1 and a representative stereo view of the experimental density map is provided in Supplementary Fig. 3a. The structure demonstrates that the N-terminal domains of two protomers form a twofold symmetrical homodimer (Fig. 2a). Each protomer consists of eight EF-hand motifs, which form an arch in a novel EF-hand arrangement. EF-hands 4–8 have evolved to form the extensive dimerization interface, whereas EF-hands 1–3 are located on the periphery (Fig. 2a). Only EF-hand 2 binds calcium, as shown by anomalous difference Fourier maps (Supplementary Fig. 3b).

**Figure 3:**
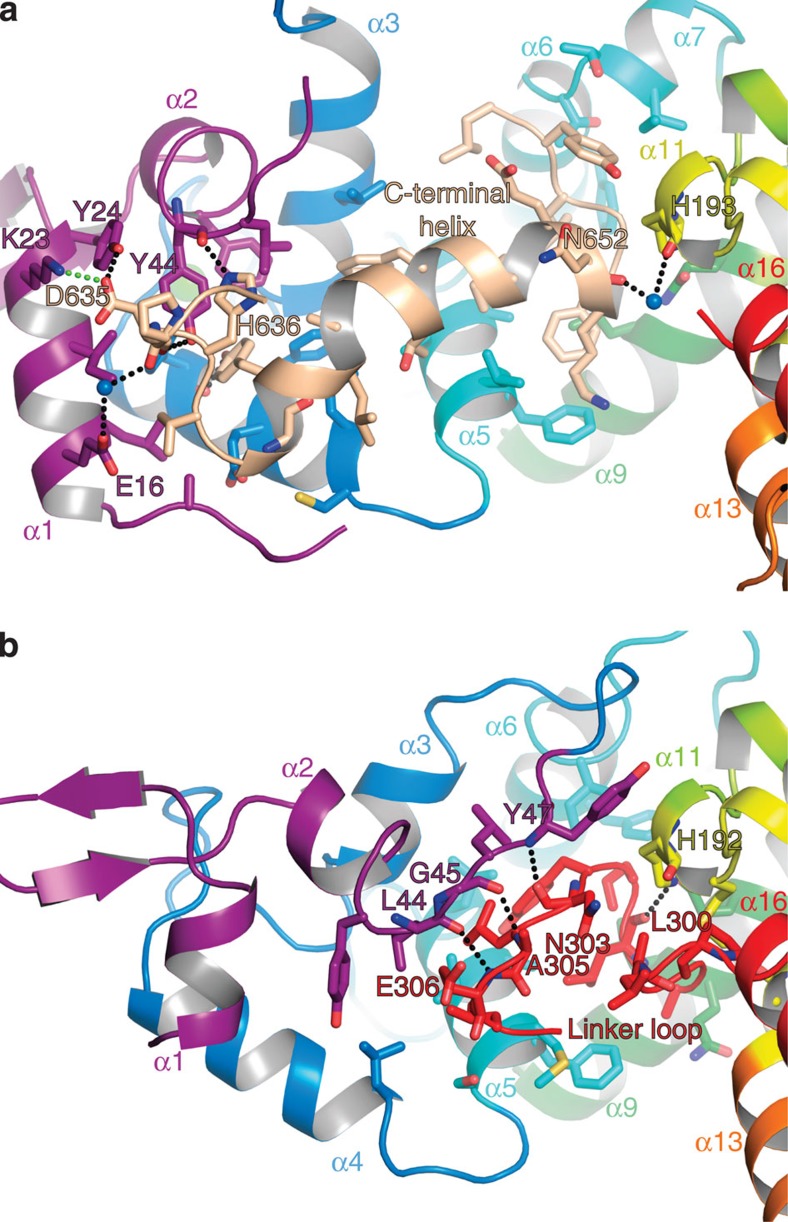
Interactions between structural elements. (**a**) Interactions between N- and C-terminal domains of citrin. Chain A is coloured according to the scheme in Fig. 1a. Hydrogen bonds between the domains are shown as black dotted lines, with donor and acceptor residues shown in stick representation. The salt-bridge interaction between residues Lys23 and Asp635 is shown as a green dotted line. Residues involved in hydrophobic contacts are shown as sticks. The water molecules involved in hydrogen-bonding networks are shown as blue spheres. (**b**) Interactions stabilizing the linker loop, which links the C-terminus of the N-terminal domain to the carrier domain, as seen in the calcium-free state of aralar. Chain B is depicted. Hydrogen bonds are shown as black dotted lines, with donor and acceptor residues shown as sticks. The orientation of the structures in the two panels is similar.

An additional helical feature was present in the electron density map, which was unambiguously assigned to residues 632–658 from the C-terminal domain (Figs 1a, 2a and 3a and Supplementary Fig. 4a). This helix is only found in the aspartate/glutamate carriers and was previously predicted to be part of the carrier domain, based on hydropathy^8^. However, the structure shows that the C-terminal helix is amphipathic and is wedged into a hydrophobic groove in the N-terminal domain (Supplementary Fig. 5a). Gly638 to Lys641 form a turn of 3_10_ helix, whereas Leu642 to Phe654 form a regular α-helix (Fig. 3a). The C-terminal domain adopts a loop conformation on either side of the helix. A large number of hydrophobic contacts involving highly conserved residues are responsible for the interaction between the N- and C-terminal domains (Supplementary Fig. 5b), and networks of aromatic residues at both ends of the C-terminal helix stabilize the interaction; Tyr640 forms an aromatic stacking arrangement with Phe78 and lies within 4 Å of Phe40, Tyr44 and Phe81, whereas Phe654 forms an edge-on stacking arrangement with Phe92. Hydrogen bonds and one salt-bridge are also involved in the interactions (Fig. 3a). Most of these are found at the N-terminal end of the C-terminal domain, where Pro634, Asp635, His636, Tyr640 and Gly639 form hydrogen bonds with Glu16 (water-mediated), Tyr24, Tyr44, Phe78 and Glu82, respectively. Asp635 also forms a salt bridge to Lys23. The C-terminal end has one water-mediated hydrogen bond between Asn652 and His193. Thus, the C-terminal helix is bound tightly to EF-hands 1 and 2, principally via its N-terminal region.

The linker of the purified fusion protein was cleaved in solution with TEV protease, and in the presence of calcium the resulting products ran as one species on a size exclusion column, indicating that the N- and C-terminal domains were bound together in a stable conformation in agreement with the structure (Supplementary Fig. 6a). The interactions between the N- and C-terminal domains are likely to be conserved in aralar, as most of the interacting residues are present except for the equivalent residue to Lys23 (Gln22), which may form a hydrogen bond with Asp634, replacing the salt bridge interaction between Lys23-Asp635 in citrin.

### Ca^2+^-bound and Ca^2+^-free structures of the aralar N-terminal domain

To obtain insight into the molecular mechanism of calcium regulation, we tried to solve a structure of the N- and C-terminal domain fusion of citrin in the calcium-free state. Crystals were soaked in the chelating agent or the fusion protein was purified in the presence of chelating agents before crystallisation trials, but calcium-free structures were not obtained. When the TEV cleavage site linker was cleaved in the presence of calcium-chelating agents EGTA and EDTA, the two domains did not separate (Supplementary Fig. 5b), likely because of the large number of interactions at the N-terminal region of the C-terminal domain that bind the C-terminal domain tightly to EF-hands 1 and 2 (Fig. 3a). We reasoned that removal of the C-terminal helix might be required to allow transition from the calcium-bound to the calcium-free state. As the N-terminal domain of citrin alone could not be expressed, we purified the N-terminal domain of aralar. Crystals were obtained in the presence of calcium as well as in the presence of chelating agents. Data sets were collected from single crystals to 2.3 and 2.4 Å, respectively (Supplementary Table 1). The structure of citrin was used to determine the structure of the calcium-bound state of the N-terminal domain of aralar by molecular replacement (Fig. 2b). In turn, the calcium-bound structure of aralar was used to determine that of the calcium-free state (Fig. 2c). The N-terminal domain of aralar shares the same overall architecture with citrin, consisting of eight EF-hands per protomer (Fig. 2b,c). EF-hands 4–8 of aralar are in a very similar position to those of citrin, whereas EF-hands 1–3 are in a similar position in the calcium-bound state, but not in the calcium-free state (Fig. 2).

In the calcium-free state of aralar, the hydrophobic groove that binds the C-terminal helix in the calcium-bound state of citrin is absent. In this region extra density was found, which was assigned to a loop of residues 296–308 (Fig. 3b). The loop links the N-terminal domain to the carrier domain (Fig. 1a) and we have named it the ‘linker loop’. A simulated annealing omit map was calculated, confirming the position of the linker loop (Supplementary Fig. 4b). There is density for parts of the linker loop in both protomers of the regulatory domain dimer, but in only one was the density sufficient for modeling. It has been postulated that this region could be part of a non-functional EF-hand^17^, but this is not the case. In the calcium-bound state of aralar, in which there is no C-terminal helix in the construct, there is no density for the linker loop, indicating that it is not bound to this region in this state. In the citrin structure, the C-terminal helix is bound, and thus binding of the linker loop to the same site would be excluded. The density map shows that the linker loop takes a different direction, but the density was insufficient for modelling.

Interactions between the linker loop and the rest of the N-terminal domain involve hydrophobic contacts and hydrogen bonds mediated predominantly by backbone amide–backbone carbonyl interactions between residues Ala296, Leu300, Asn303, Ala305 and Glu306, and residues Pro294, His192, Tyr47, Gly45 and Leu44, respectively (Fig. 3b).

### Molecular basis of calcium binding

As calcium-bound and calcium-free structures were available, we could analyse the molecular nature of state-dependent calcium binding. On the basis of the relative angles of the EF-hand α-helices, EF-hands 4, 5 and 6 are in a closed conformation, whereas EF-hands 1, 2, 3, 7 and 8 are in an open conformation in all structures^33^. However, in the calcium-bound state only EF-hand 2 binds calcium, as shown by anomalous difference Fourier maps of citrin (Supplementary Fig. 3b).

**Figure 4:**
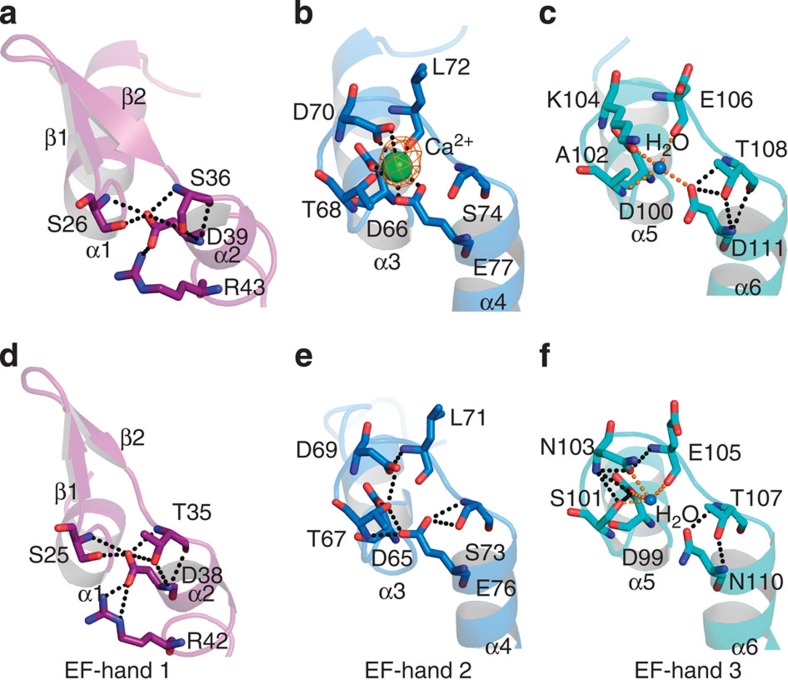
Calcium binding sites in the calcium-bound and calcium-free state of the N-terminal domain. (**a**) S100-like EF-hand 1 in the calcium-bound state of citrin with a short anti-parallel beta-sheet in the loop region. (**b**) EF-hand 2 in the calcium-bound state of citrin with residues involved in the pentagonal mono-pyramidal coordination of calcium. Calcium is shown as a green sphere, with an anomalous difference Fourier map shown as an orange mesh contoured at a 5.0 σ-level. (**c**) EF-hand 3 in the calcium-bound state of citrin with residues involved in hydrogen bonds and the coordination of water (blue sphere). (**d**) S100-like EF-hand 1 in the calcium-free state of aralar (**e**) EF-hand 2 in the calcium-free state of aralar (**f**) EF-hand 3 in the calcium-free state of aralar. Hydrogen bonds are shown as black dotted lines and hydrogen bonds involved in the coordination of water molecules in orange dotted lines.

In all structures, EF-hand 1 differs from canonical EF-hands and resembles those found in the S100 protein family^34^. The calcium-binding loop of EF-hand 1 has an insertion of three extra amino acids and contains a short two-stranded antiparallel β-sheet (Fig. 4a,d). EF-hand 1 does not bind calcium in either of the calcium-bound states (Supplementary Fig. 3b) in contrast to earlier predictions^17^, and thus it is not directly involved in the regulation of the aspartate/glutamate carrier.

In the calcium-bound state of citrin, EF-hand 2 binds calcium (Supplementary Fig. 3b), which is coordinated in a pentagonal monopyramidal arrangement (Fig. 4b). The distances of calcium to the six ligands are between 2.3–2.4 Å, similar to the canonical coordination geometry^35^. In the calcium-bound state of aralar, EF-hand 2 binds calcium in a pentagonal bipyramidal manner with the seventh contact point provided by water. In the calcium-free state of aralar no calcium ion is present in EF-hand 2 (Fig. 4e). Interestingly, release of calcium from EF-hand 2 did not change the relative orientations of helices α3 and α4, as frequently observed in EF-hand-containing proteins. Hence, the calcium-free state has a ‘preformed’ EF-hand, ready for calcium binding, potentially reducing the entropic cost. This arrangement has been observed for other EF-hands and might explain the high affinity for calcium^34^.

In citrin, EF-hand 3 was predicted to be non-functional^17^ and indeed no calcium was present in the site (Fig. 4c and Supplementary Fig. 3b). Instead EF-hand 3 binds an ordered water molecule and is stabilized by multiple hydrogen bonds. A similar arrangement is found in the calcium-bound and calcium-free states of aralar (Fig. 4f). It has been suggested previously that EF-hand 3 of aralar binds calcium^17^, but the molecular arrangement shows that this is not the case. The 12th residue of the calcium chelation loop has an asparagine residue rather than a negatively charged aspartate or glutamate residue, which is required for the bidentate side-chain ligation of calcium^34^.

EF-hands 4–8 do not bind calcium either, but they have evolved to form an extensive dimerization interface. The helical arrangement of EF-hands 4, 5 and 6 is in a closed conformation, whereas 7 and 8 are in an open configuration. Similar to calmodulin^36^, the exiting helix of EF-hand 4 is fused to the entering helix of 5, and likewise EF-hand 6 is fused to EF-hand 7 (Figs 1a and 2a,b). It has been suggested that EF-hand 5 of aralar chelates calcium^17^, but it is in a closed conformation in all three structures and is involved in dimerization. Thus, EF-hands 4–8 are not involved in calcium binding.

### EF-hands 4–8 form the static unit of the N-terminal domain

All three structures are homodimeric (Supplementary Fig. 7), consistent with the SEC-MALLS data (Fig. 1b–e). The structure of the dimerization interface, formed by EF-hands 4–8 of the N-terminal domain, is very similar in all three crystal forms (RMSD of the calcium-bound state of aralar and citrin: 0.93 Å, RMSD of the calcium-free state of aralar and citrin: 0.83 Å). The extensive dimerization interface in citrin covers a buried surface area of 2,200 Å^2^ per monomer (14% of the solvent-accessible surface), and is stabilized by 17 hydrogen bonds and two salt bridges (Supplementary Fig. 7a). In the calcium-bound and calcium-free states of aralar, the dimerization interface covers a buried surface area of ~1,800 Å^2^ (12%) and 2,000 Å^2^ (15%) per protomer, respectively. In the calcium-bound state of aralar the interaction is stabilized by 14 hydrogen bonds and six salt bridges (Supplementary Fig. 7b), whereas in the calcium-free state there are 17 hydrogen bonds and six salt bridges (Supplementary Fig. 7c). The hydrogen bonds formed by side-chains are conserved between the calcium-bound states of citrin and aralar (Supplementary Fig. 7a,b).

A comparison of the dimerization interfaces of the calcium-bound and calcium-free states of aralar reveal only two changes in the hydrogen bonds formed by side-chain interactions (Supplementary Fig. 7b,c). In the calcium-bound state residue Gln152 (Gln153 in citrin) forms hydrogen bonds to residues Thr264 (Thr265) and Glu267 (Glu268). In the calcium-free state Gln152 forms a hydrogen bond to Thr264, but the interaction to Glu267 is absent as a result of subtle movements. The other difference is that in the calcium-free state a hydrogen bond is formed between Asn228 of EF-hand 6 and Gln262 of EF-hand 7 of two protomers. In the calcium-bound state there is no density for the side-chain of Gln262, indicating that it is not interacting. Instead, the Asn228 side chain forms a hydrogen bond to the backbone nitrogen of Gln262 within a protomer. Overall, the analysis indicates that the dimer formed by EF-hands 4–8 is largely static except for small movements in EF-hands 6–8 in response to calcium binding.

### EF-hands 1–2 form the mobile unit of the N-terminal domain

**Figure 5:**
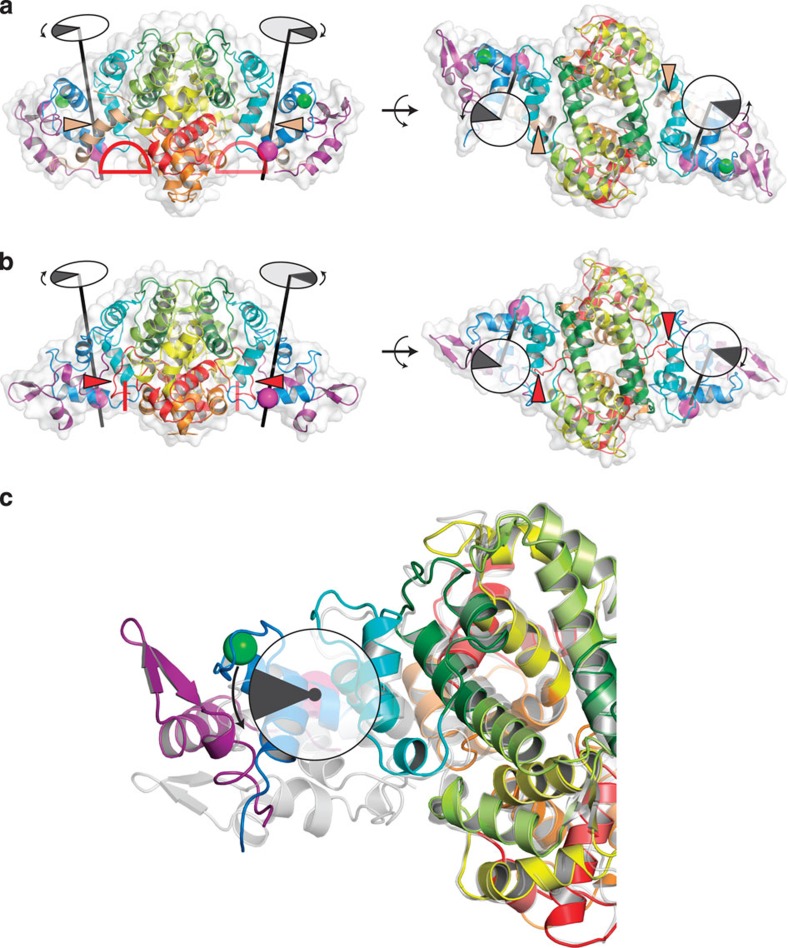
Movements of the N-terminal domain of the aspartate/glutamate carrier in response to calcium binding. (**a**) The calcium-bound state of the N-terminal domain of citrin with the C-terminal helix bound. The C-terminal helix is shown in a cartoon representation and is coloured wheat. The bound calcium ions are shown as green spheres. The red semi-circles indicate the opening of a vestibule. (**b**) The calcium-free state of the N-terminal domain of aralar (chain B) with the linker loop in red. The red lines indicate the closure of the vestibule. The cartoon representations are coloured as in Fig. 1a. The conformational change between states involves a rotation of EF-hands 1–2 relative to the static domain of EF-hands 4–8. The rotations are shown by circles with black sectors and arrows marking the angle and direction of rotation, and a black line marking the rotation axis. Magenta spheres highlight the bending region corresponding to residues 82 (aralar) and 83 (citrin). The C-terminal helix and the linker loop are indicated with wheat and red coloured arrow heads, respectively. (**c**) A view down the rotation axis, showing the conformational change between calcium-bound citrin and calcium-free aralar. Citrin is shown as a cartoon, coloured as in Fig. 1a, with the C-terminal domain removed for clarity. The calcium-free state of aralar is shown as a grey cartoon.

Superimposition of EF-hands 4–8 of the calcium-bound state of citrin and the calcium-free state of aralar reveals a striking conformational change in which EF-hands 1–2 are involved in a rigid-body rotation (Fig. 5 and Supplementary Movie 1). The transition from the calcium-bound state to the calcium-free state involves a rotation of the unit of EF-hands 1–2 by ~47 degrees (Fig. 5c), with the rotation axis oriented between EF-hands 2 and 3. The bending region, corresponding to residues 82 (aralar) or 83 (citrin), is located on the C-terminal side of helix α4, close to the loop linking EF-hands 2 and 3. Once closed, the loop between EF-hands 1 and 2 (Tyr47, Ser52, Asn51, Asn53 and Lys55) interacts with residues in the loop between EF-hands 3 and 4 (Thr118, Ile119, His121) and the loop between EF-hands 4 and 5 (Thr188 and Ser191, which lie at a turn of 3_10_ helix). As a consequence, a cavity in the regulatory domain closes (Fig. 5a,b). Comparison of calcium-free and calcium-bound aralar structures confirms the nature of the conformational change with EF-hands 1–2 rotating by 42 degrees, and with the rotation axis and bending region lying in a similar position.

## Discussion

**Figure 6:**
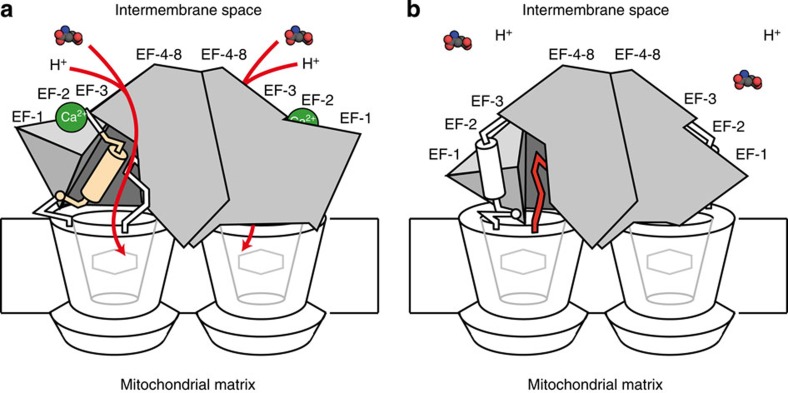
Proposed mechanism of calcium regulation of the mitochondrial asparate/glutamate carrier. (**a**) The calcium-bound state of the aspartate/glutamate carrier dimer. On calcium binding the mobile sub-domain of EF-hands 1–2 moves to open up a hydrophobic groove in which the α-helix of the C-terminal domain (wheat) binds. Glutamate and a proton enter the carrier domain to trigger the conformational changes required for the translocation of the substrates. (**b**) The calcium-free state of the aspartate/glutamate carrier dimer. In the absence of calcium, the mobile sub-domain of EF-hand 1–2 closes the hydrophobic groove and the C-terminal helix is no longer bound. Instead the linker loop (red) binds to close the gap. The conformational changes may limit access of the substrates to the carrier domain. The substrate-binding site is shown as a hexagon.

Here we have presented structures of the regulatory domains of the mitochondrial aspartate/glutamate carriers citrin and aralar. In both cases the regulatory domain is the site of calcium regulation, which leads to the stimulation of the malate-aspartate shuttle in the presence of calcium and a return to basal activity of the shuttle in the absence of calcium^6^,^17^,^18^,^19^,^20^. The SEC-MALLS data (Fig. 1) and structures (Fig. 2) show that the carriers form homodimers mediated only by the N-terminal domains. The striking aspect is that many EF-hand folds of this domain have evolved away from their canonical calcium-binding functions to provide a platform for dimerization. The carrier domains alone do not dimerize and thus they are likely to function independently, held together by the N-terminal domains. The twofold symmetry axis of the N-terminal domain dimer is likely to be perpendicular to the membrane, meaning that only two possible orientations relative to the carrier domains need to be considered. There are several arguments that favour the orientation in which the ridge formed by EF-hands 1–3 is facing the water phase (Fig. 6). First, the ridge is highly negatively charged making it unlikely to face the membrane. Second, the binding site in EF-hand 2 would be accessible to calcium ions in the water phase. Third, in the calcium-bound state a cavity opens in the regulatory domain, which in the proposed orientation would lie proximal to the carrier domain, acting as a vestibule to allow access of the substrates. Fourth, in the absence of calcium the mobile unit of EF-hands 1–2 moves downwards as well as inwards (Fig. 5). It is most likely that this motion is directed towards the carrier domain.

There are no large movements of EF-hands 4–8 when the calcium-bound and calcium-free structures are compared, indicating that this unit is largely static. Dimerization might be required to form a rigid framework against which the conformational changes involving the mobile unit can take place (Fig. 5). Our structures demonstrate that EF-hands 1 and 2 form the calcium-responsive mobile unit, which is consistent with several other observations. Deletion of EF-hand 1 in citrin was shown to result in a complete loss of calcium-binding capacity^10^, whereas specific mutations introduced in EF-hands 1 and 2 of aralar abolished the stimulation of the malate–aspartate shuttle by calcium^20^. Both aralar and citrin bind calcium only via EF-hand 2, which has the correct molecular arrangement for calcium coordination. Thus, structurally there is no difference between aralar and citrin with respect to calcium regulation.

The structure of citrin shows that in the calcium-bound state the C-terminal helix is bound to the N-terminal domain. Since it was a fusion protein, one could argue that the observed binding of the C-terminal helix to the N-terminal domain is an artefact. There are several compelling arguments against this notion. First, the unstructured flexible loop (not resolved in the crystal structures) that links the carrier domain to the C-terminal helix is 28-amino-acids long (a distance of about 100 Å). Thus, the C-terminal helix is not restrained by the fusion and would be free to bind anywhere on the surface of the N-terminal domain. However, the helix was clearly resolved in the electron density map (Supplementary Fig. 4a), showing that it was binding consistently to the same site with high occupancy. Second, many conserved interactions are involved in the binding (Supplementary Fig. 5b), and the C-terminal domain remains bound to the N-terminal domain after cleavage at the TEV protease site (Supplementary Fig. 6). Third, the groove is composed of conserved hydrophobic residues (Supplementary Fig. 5a,b), which would be highly unusual for a water accessible surface, but entirely compatible with binding the amphipathic C-terminal helix (Supplementary Fig. 5c). The properties of the hydrophobic groove and amphipathic helix point therefore to an important functional feature. The calcium-dependent binding of an amphipathic helix to a hydrophobic groove is used for the binding of extrinsic target proteins by calmodulin^37^ and the S100 proteins^38^,^39^, and it appears that a similar mechanism operates here. In the case of the aspartate/glutamate carriers, the functional elements of this conserved mechanism have been separated by insertion of the carrier domain, providing a new application of calcium regulation by EF-hand-containing proteins.

In the calcium-free state of aralar the linker loop is bound in the same area (Fig. 3b and Supplementary Fig. 4b). In the calcium-bound state of citrin weak density for the linker loop is found in a different position, indicating that the loop is mobile and follows a different path.

Combining all of these elements we propose a mechanism of calcium regulation of mitochondrial aspartate/glutamate carriers. At increased calcium levels, the regulatory domain undergoes calcium-dependent conformational changes in which EF-hands 1–2 move to expose a hydrophobic binding site for the binding of the amphipathic α-helix of the C-terminal domain, excluding the binding of the linker loop (Fig. 6a). As a consequence, a vestibule opens through which a glutamate and proton can enter the carrier domain to trigger the conformational changes required for the translocation of the substrates. The calcium-induced interaction of the N-terminal domain with the C-terminal helix could therefore result in the upregulation of transport. In the absence of calcium, the mobile unit of EF-hands 1–2 closes the hydrophobic groove (Fig. 6b). The role of the linker loop is tentative, especially since its sequence is not strictly conserved, but it could function to seal the remaining gap. In the calcium-free state the C-terminal helix stays bound to the N-terminal domain (Supplementary Fig. 6), poised to bind again to the hydrophobic site in the calcium-bound state. As a consequence of the motion, the vestibule closes, which may prevent substrates from entering the carrier domain. The structures were generated in the presence of high concentrations of calcium or chelating agents, and the protomers are in the same state. However, it is possible that under physiological conditions the two protomers of the dimer could act independently. The model we propose is an on–off mechanism for the protomer, which is compatible with observations of physiological activity describing a basal transport activity^6^,^17^,^18^, as they deal with regulation of carriers on the population level.

The urea cycle is dependent on the export of aspartate from the mitochondrion by the aspartate/glutamate carrier. Mutations in the *SLC25A13* gene lead to metabolic diseases caused by an impaired urea cycle, collectively called citrin deficiency. There are three age-dependent disease phenotypes—neonatal intrahepatic cholestasis caused by citrin deficiency (NICCD) in newborns, failure to thrive and dyslipidemia caused by citrin deficiency (FTTDCD) in young children and citrullinemia type 2 (CTNL2) with childhood to adult onset^40^. Citrin deficiency is considered a panethnic disorder with the highest estimated frequencies of 1 in 17,000 for NICCD to 1 in 230,000 for CTNL2 in the East Asian population^41^,^42^. Symptoms range from cholestasis and multiple aminoacidemia in NICCD to recurrent encephalopathy and hyperammonemia in CTNL2 (ref. 43). The NICCD symptoms often diminish by the first year, but in some cases patients develop CTNL2 later in life^43^. CTNL2 is effectively treated by liver transplantation, but, if left untreated, brain edema a few years later results in death^43^.

**Figure 7:**
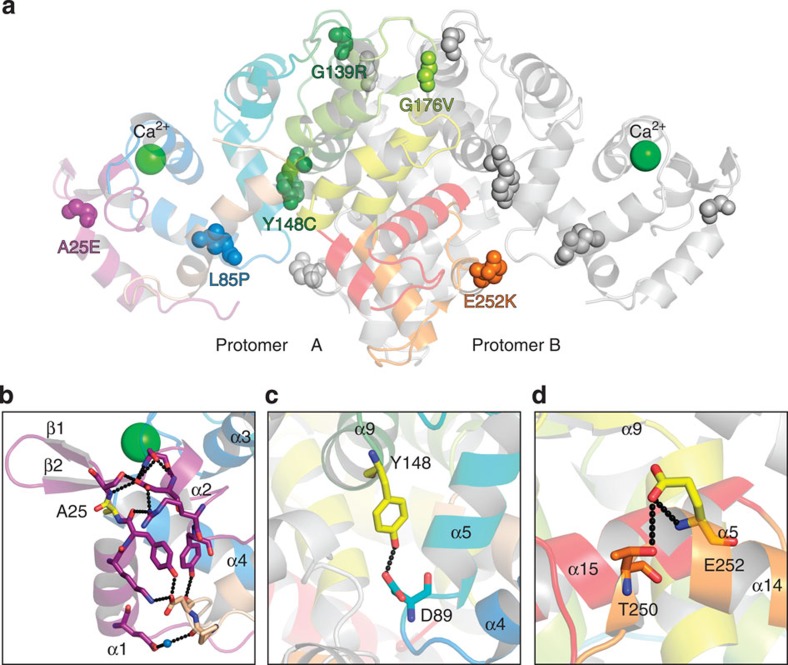
Missense mutations in the N- and C-terminal domains of citrin causing citrin deficiency. (**a**) Six missense mutations found in the N-terminal domain of citrin. The overall structure of the regulatory domain of citrin is depicted and is coloured according to Fig. 1a. The residues of the missense mutations are depicted in sphere representation. Bound calcium ions are shown as green spheres. (**b**) Key interactions within EF-hand 1 and with the C-terminal helix near Ala25. Residues forming key interactions are shown in stick representation. EF-hand 1 and the C-terminal helix are shown in purple and wheat, respectively. A water molecule mediating an interaction between EF-hand 1 and the C-terminal helix is shown as a blue sphere. Ala25 is shown in a yellow stick representation. (**c**) Interaction of Tyr148 with the conserved Asp89. Tyr148 and Asp89 are shown in stick representation and coloured in yellow and cyan, respectively. (**d**) Interaction of Glu252 with conserved Thr250. Glu252 and Thr250 are shown in stick representation and coloured in yellow and orange, respectively. Hydrogen bond interactions are shown as black-dotted lines.

Mutations causing CTNL2 in *SLC25A13* were first identified 15 years ago and individuals are homozygotes or compound heterozygotes for citrin deficiency^9^,^44^. Since then, 83 mutations causing citrin deficiency have been identified, of which six missense mutations are found in the regulatory domain of citrin^40^,^45^,^46^. We have mapped all six onto the structure of the regulatory domain of citrin (Fig. 7a), providing a structural framework for the interpretation of the effect of these mutations.

Residue Ala25 in EF-hand 1, which is mutated to glutamate in NICCD, is conserved among aspartate/glutamate carriers and is part of an extensive network of polar interactions involving EF-hand 1 as well as contacts between the N- and C-terminal domains (Fig. 7b). An introduction of a large and negatively charged residue may affect this network. Western blot analysis of fibroblast cultures of patients carrying the A25E mutation on both alleles showed that there was no difference in protein levels^41^, indicating that this mutation affects function rather than biogenesis of the protein.

Leu85 is part of the exiting helix of EF-hand 2 and in one case has been mutated to proline (NICCD). Sequence alignments show a preference for leucine at that position, but isoleucine and methionine are also tolerated. A western blot analysis of a liver biopsy of the patients carrying a compound heterozygous mutation (E601K and L85P) demonstrated that citrin was absent^47^. The introduction of a proline residue in helix 4 of EF-hand 2 could truncate the helix, which is likely to be detrimental to the protein structure.

Residues Gly139 and Gly176 are conserved and present in the dimerization interface (Fig. 7a). Mutations G139R (NICCD) and G176V (NICCD) introduce a large positively charged or hydrophobic residue into a tightly packed dimerization interface, which might lead to disruption of the dimer, which according to our model will significantly affect activity.

Tyr148, which is mutated to cysteine in CTNL2, forms a hydrogen bond with conserved Asp89 in the loop region between EF-hands 2 and 3 (Fig. 7c). In the calcium-free state of aralar Tyr147 (the equivalent residue to Tyr148) forms an additional hydrogen bond to the backbone carbonyl group of Asp88. The Y148C mutation would eliminate the hydrogen bond and might affect both states. Glu252, which in NICCD is mutated to lysine, forms a hydrogen bond to its own backbone nitrogen and to the hydroxyl group of conserved Thr250 (Fig. 7d). E252K would disrupt the hydrogen bond and may lead to the loss of structural integrity in this region. With these structures we begin to understand the molecular basis of these mitochondrial pathologies.

## Methods

### Purification of Ca^2+^-bound N- and C-terminal citrin fusion

The fusion of the N- and C-terminal domains of citrin was constructed by joining the N-terminal domain (residues 2–319), containing an N-terminal octa-histidine tag and Factor-Xa cleavage site, to the C-terminal domain (residues 613–675) of citrin (Uniprot: Q9UJS0) via a TEV protease cleavage site (ENLYFQG). The gene was codon-optimized for expression in *Lactococcus lactis* by GenScript (US) and cloned into the *L. lactis* expression vector pNZ8048. The vector was transformed into electrocompetent *L. lactis* NZ9000 using established methods^48^. Transformants were selected on 5 μg ml^−1^ chloramphenicol-containing SM17 agar plates, and the strains were stored in M17 medium plus 10% (v/v) glycerol at −80 °C.

A 2-l starter culture of the *L. lactis* expression strain was grown in M17 medium supplemented with 1% (w/v) glucose and 5 μg ml^−1^ chloramphenicol at 30 °C overnight and used to inoculate an Applikon bioreactor with 100 l of the same medium. Expression of the proteins was induced at an A_600_ of 0.5 with a 1:1,000 dilution of spent medium of strain NZ9700 producing Nisin A. After induction the incubation temperature was held at 30 °C for 4 h and then lowered to 20 °C overnight. Cells were washed in 1 × TBS and harvested by centrifugation. Cell pellets were stored at −80 °C until further use.

*L. lactis* cells (~35 g) expressing the citrin N- and C-terminal fusion protein were homogenized in lysis buffer (50 mM Hepes, pH 7.5, 500 mM NaCl, 5% (v/v) glycerol, 10 mM imidazole) with a Dounce homogenizer to a final volume of 100 ml, and then supplemented with 1 mM phenylmethylsulfonylfluoride (PMSF, Sigma), 40 μg ml^−1^ DNase (Sigma) and 10 μg ml^−1^ RNase (Sigma). The cell suspension was passed once through a pre-chilled cell disrupter (Constant Cell Disruption System 2.2 kW Z Plus) at 33 kpsi. The cell lysate was clarified by a low-speed centrifugation step (4,000 *g*, 15 min, SLA1500 rotor (Thermo Scientific)) and then by a high-speed centrifugation step (40,000 *g*, 60 min, SS34 rotor (Thermo Scientific)). The sample was loaded onto a pre-equilibrated 2 ml Nickel Sepharose High Performance column (GE Healthcare) at a flow-rate of 1 ml min^−1^ with an ÄKTA prime (GE Healthcare). Unbound proteins were removed by washing with lysis buffer containing 100 mM imidazole and bound proteins were eluted step-wise with lysis buffer containing 150, 200 or 300 mM imidazole. The 200 and 300 mM imidazole elution fractions were pooled, supplemented with 1 mM dithiothreitol (DTT, Sigma) and concentrated to a volume of ~1.5 ml in a 10 kDa MWCO centrifugal concentrator (Millipore). The sample was injected at 1 ml min^−1^ onto a Superdex 200 HiLoad 16/60 120 pg column (GE Healthcare) equilibrated with SEC buffer (10 mM Hepes, pH 7.5, 150 mM NaCl, 1 mM DTT). Peak fractions were pooled and concentrated in a 30 kDa MWCO centrifugal concentrator (Millipore) to ~7 mg ml^−1^ for crystallization trials and ~10 mg ml^−1^ for SEC-MALLS analysis. All purification steps were carried out at 4 °C. The protein concentration was determined by bicinchoninic acid (BCA) assay (Thermo Scientific). The purified proteins were flash frozen and stored in liquid nitrogen.

### Crystallization of Ca^2+^-bound N- and C-terminal citrin fusion

Initial crystallisation trials were performed by using the vapour diffusion sitting drop method at 22 °C with commercially available crystallisation screens (Molecular Dimensions). For this purpose the fusion protein was supplemented with 1 mM CaCl_2_ and 200+200 nl drops were set up with a Mosquito dispensing robot (TTP Labtech). Crystals appeared the next day in 0.2 M sodium malonate, pH 7.0 and 20% (w/v) PEG 3350 (Molecular Dimensions JCSG+ HT-96 G06). Crystals were cryo-protected with 25% (v/v) PEG 400 and harvested with MiTeGen cryo loops after 8 days. For the generation of derivatives, crystals were transferred to mother liquor containing HgCl_2_, ethyl mercury thiosalicylate (EMTS), K_2_PtCl_4_ or KAu(CN)_2_. Mercury compounds were used at a concentration of 1 or 5 mM, whereas platinum and gold compounds were used at a concentration of 1 mM. Soaking times varied from 2 to 24 h. Crystals were back soaked in mother liquor without heavy metal before being cryo-protected and cooled in liquid nitrogen.

### Structure determination of Ca^2+^-bound N- and C-terminal citrin fusion

Data sets used for experimental phasing were collected at the ID23-1 beamline at the European Synchrotron Radiation Facility (ESRF, Grenoble, France)^49^, using a 30 μm X-ray beam. The native data set used for refinement (Native 1) was collected at the ID23-2 microfocus beamline at ESRF, using a 10-μm X-ray beam^50^. All data sets were collected from cryo-cooled crystals at 100 K, using helical oscillation strategies to minimize the effect of radiation damage. Intensities were integrated using the program XDS^51^,^52^, and merged and scaled using the program Aimless^53^. Data collection statistics are shown in Supplementary Table 1.

Phases were determined using the MIRAS technique with data collected from six crystals: one native and five derivatives. The derivative crystals came from the following soaking procedures: 24 h in 1 mM EMTS, 24 h in 5 mM EMTS, 2 h in K_2_PtCl_4_, 2 h in KAu(CN)_2_ and 24 h in KAu(CN)_2_. Native and derivative data sets were scaled using Scaleit^54^, initial sites were found with ShelxC/D^55^, and phases were calculated and refined with Sharp, as implemented in autoSHARP^56^. The figure-of-merit before density modification was 0.322 for acentric reflections and 0.439 for centric reflections. Density modification was carried out using Solomon in autoSHARP, resulting in maps showing excellent connectivity and clear density for many side-chains. Buccaneer^57^,^58^ was used for initial model building, producing a model 85% complete by residues built. The model was completed by iterative cycles of model building in Coot^59^, and refinement in Refmac^60^ and Phenix.refine^61^, to a final *R*=19.3%, *R*_free_=22.7%. Molprobity^62^ model validation shows that 98.3% of protein residues are in the favoured region of the Ramachandran plot with 0% outliers. The final model has a Molprobity clashscore of 1.94 and an overall Molprobity score of 0.96.

Data set Native 2 was collected from a single crystal at 100 K using 6.5 keV X-rays at beamline I24, Diamond Light Source (Harwell, UK). Phases were determined by molecular replacement using Phaser^63^, with non-protein atoms excluded, followed by refinement with jelly-body restraints in Refmac. Anomalous difference Fourier maps were calculated in Coot.

### Purification of Ca^2+^-bound N-terminal domain of aralar

Initially, a fusion of the N- and C-terminal domains of aralar was designed by using residues 2–317 of the N-terminal domain and residues 611–678 of the C-terminal domain of aralar (Uniprot: O75746) linked via a TEV protease cleavage site (ENLYFQG). The gene was codon-optimized for expression in *L. lactis* by GenScript (US). The gene encoding the N-terminal domain of aralar (residues 2–311) with an N-terminal octa-histidine tag and a Factor Xa cleavage site was amplified from the fusion construct by polymerase chain reaction (PCR) and cloned into the *L. lactis* expression vector pNZ8048. The vector was transformed into electrocompetent *L. lactis* NZ9000, and transformants were selected according to established methods^48^. The growth, induction, storage and cell homogenization conditions for the production of the N-terminal domain of aralar were similar to that of the citrin fusion protein (see above).

The clarified sample was loaded onto a pre-equilibrated 1 ml Nickel Sepharose High Performance column at a flow rate of 1 ml min^−1^ with an ÄKTA prime FPLC system. Non-specifically bound proteins were removed with lysis buffer containing 100 mM imidazole and bound proteins were eluted with 500 mM imidazole. The eluate was diluted with lysis buffer fivefold and concentrated to 1 ml using a 30 kDa MWCO centrifugal concentrator (Millipore). The tag was removed with 25 μg Factor Xa (New England Biolabs) in the presence of 5 mM CaCl_2_ and 5 mM DTT at 4 °C overnight. The sample was injected onto a Superdex 200 HiLoad 16/60 120 pg column (GE Healthcare) at 1 ml min^−1^ with SEC buffer (20 mM Hepes, pH 7.5, 150 mM NaCl, 5 mM CaCl_2_). Peak fractions were pooled and concentrated to ~10 mg ml^−1^ in a 30 kDa MWCO centrifugal concentrator (Millipore). The protein concentration was determined with the NanoDrop ND-1000 (Thermo Scientific) using parameters determined with ExPasy ProtParam^64^. Purified protein was flash frozen and stored in liquid nitrogen.

### Crystallization of Ca^2+^-bound N-terminal domain of aralar

Crystallization trials with the calcium-bound N-terminal domain of aralar were performed by using the vapour diffusion sitting drop method at 22 °C with commercially available crystallization screens (Molecular Dimensions). For crystallization trials 200+200 nl drops were set up with a protein at a final concentration of 10 mg ml^−1^ using a Mosquito dispensing robot. Crystals appeared on the next day in 0.1 M MIB, pH 8.0 and 25% (w/v) PEG 1500 (Molecular Dimensions PACT HT-96 screen, condition B05). They were cryo-protected with 25% (v/v) PEG 400, harvested with a 200-μm loop and cryo-cooled in liquid nitrogen after 4 days.

### Structure determination of Ca^2+^-bound N-terminal domain of aralar

A native data set was collected at beamline I03, Diamond Light Source, from a single crystal at 100 K using a 20 μm X-ray beam (13.8 keV energy) and a helical data collection strategy. Intensities were integrated using XDS, and merged and scaled using Aimless. Data collection statistics are shown in Supplementary Table 1.

Phases were determined by molecular replacement using a search model produced by Phenix.Sculptor^65^ from the structure of the citrin N- and C-terminal fusion. Phaser found a solution with one copy in the asymmetric unit and a low final log-likelihood gain of 55. Refinement of the solution in Refmac with jelly-body restraints led to a significant drop in the R-factors (*R* dropped from 54.0 to 35.1%, and *R*_free_ from 54.6 to 40.6%), and the resulting maps showed density for side chains excluded from the MR search model. Rebuilding of the model was initiated in Phenix.autobuild^66^, using Resolve^67^ for density modification and Buccaneer for model building. Cycles of model building in Coot and refinement in Phenix.refine led to a final model with *R*=23.1% and *R*_free_=27.4%. The model was validated with Molprobity. The Ramachandran plot has 97.7% of protein residues in the favoured region and 0% outliers. The final model has a Molprobity clashscore of 0.97 and an overall Molprobity score of 0.86.

### Purification of Ca^2+^-free N-terminal domain of aralar

Purification of the N-terminal domain of aralar in the calcium-free state was identical to the purification procedure of the calcium-bound state until the tag removal step. The eluted sample was incubated for 15 min with 50 mM EDTA, pH 8.0 and 50 mM EGTA, pH 7.5. EDTA and EGTA were removed from the sample by diluting in lysis buffer followed by ultrafiltration with a 30-kDa MWCO centrifugal concentrator. The sample was concentrated to 1 ml and 50 μg Factor Xa (New England Biolabs) was added for tag removal overnight. 10 mM EDTA and 10 mM EGTA were added to the sample and incubated for 15 min on ice. The sample was injected onto a Superdex 200 HiLoad 16/60 120 pg column at 1 ml min^−1^ with SEC buffer (20 mM Hepes, pH 7.5, 150 mM NaCl, 10 mM EDTA, pH 8.0 and 10 mM EGTA, pH 7.5). Peak fractions were pooled and concentrated to 7.5 mg ml^−1^ in a 30-kDa MWCO centrifugal concentrator. The protein concentration was determined using the NanoDrop ND-1000 (Thermo Scientific) with parameters determined using ExPasy ProtParam^64^. Purified protein was flash frozen and stored in liquid nitrogen.

### Crystallization of Ca^2+^-free N-terminal domain of aralar

Crystallization trials were performed using the vapour diffusion sitting drop method at 22 °C with commercially available crystallisation screens (Molecular Dimensions). For trials 200+200 nl drops were set up at a final concentration of 8 mg ml^−1^ with a Mosquito dispensing robot. A single crystal appeared on the next day in 0.3 M sodium acetate, 0.1 M Tris, pH 8.5, 8% (w/v) PEG 20000, and 8% (v/v) PEG 550 MME (Molecular Dimensions Clear Strategy Screen I HT-96, condition H07), which was cryo-protected with 25% (v/v) PEG 400, harvested with a 75 μm loop and cryo-cooled in liquid nitrogen after 7 days.

### Structure determination of Ca^2+^-free N-terminal domain of aralar

A native data set was collected at beamline I24, Diamond Light Source, from a single crystal at 100 K using a 20 μm X-ray beam (12.7 keV energy) and a helical data collection strategy. Intensities were integrated using XDS, and merged and scaled using Aimless. Data collection statistics are shown in Supplementary Table 1.

Phases were initially determined by molecular replacement using the calcium-bound aralar structure, truncated at residue 92 (removing EF-hands 1 and 2) and with non-protein atoms removed, as a search model. Phaser found a single solution with two copies in the asymmetric unit and a final log-likelihood gain of 1226. Refinement of the MR solution in Refmac with jelly-body restraints led to a significant drop in R-factors (*R* dropped from 44.7 to 36.6%, and *R*_free_ from 44.1 to 39.6%). The conformation of the N-terminal region of the search model, including EF-hand 3, was clearly different compared with the calcium-bound state, and difference density was observed for EF-hands 1 and 2, particularly for chain B. Phenix.autobuild was used to rebuild and extend the preliminary model, using Resolve for density modification and Buccaneer for model building. The resulting model included EF-hands 1 and 2 for chain B, with *R*=27.1% and *R*_free_=33.4%. Iterative cycles of model building in Coot and refinement in Phenix.refine resulted in a final model with *R*=24.52%, *R*_free_=29.66%. The electron density maps of the calcium-free state contain a minor residual density in EF-hand 2, which is insufficient for a calcium ion. Refinement with calcium modelled in this density leads to an unacceptably high B-factor (520 Å^2^ compared to 80–100 Å^2^ for the surrounding atoms), instead a water molecule or a small ion, such as sodium, is likely to bind. Chains A and B of the calcium-free structure have different crystal contacts, yet adopt the same conformation (Supplementary Fig. 3c), indicating that the proposed conformational changes are not the consequence of crystal packing. The linker loop is modelled only in chain B. The model was validated using Molprobity. The Ramachandran plot has 97.7% of protein residues in the favoured region and 0.4% outliers, and the model has a Molprobity clashscore of 3.26 and an overall Molprobity score of 1.19.

The structural morph shown in the Supplementary Movie was produced using the UCSF Chimera package from the Resource for Biocomputing, Visualization and Informatics at the University of California, San Francisco (supported by NIH P41 RR-01081)^68^. The dimerization interfaces were analysed with PISA^69^. Conformational changes between states were analysed with DynDom^70^. The rendered images were generated with the PyMOL Molecular Graphics System, Schrödinger, LLC.

### SEC-MALLS

SEC-MALLS was performed with a Superdex 200 10/300 GL column (GE Healthcare) or a Superose 6 10/300 GL column on an ÄKTA Explorer (GE Healthcare) coupled in-line with a light scattering detector (Dawn HELEOSII, Wyatt Technologies) and a refractive index detector (Optilab T-rREX, Wyatt Technologies). The N-terminal domain of aralar, the N- and C-terminal fusions of aralar and citrin, were injected onto the Superdex 200 10/300 GL column equilibrated with 20 mM Hepes, pH 7.5, 150 mM NaCl and 5 mM CaCl_2_. Citrin was injected onto the Superose 6 10/300 GL column equilibrated with 20 mM Hepes, pH 8.0, 150 mM NaCl, 0.002% (w/v) LMNG and 0.004 mg ml^−1^ TOCL. The carrier domain of citrin and Aac2p were injected onto a Superose 6 10/300 GL column equilibrated with 20 mM Hepes, pH 8.0, 150 mM NaCl, 2.5 mM DTT, 0.03% (w/v) DDM and 0.03 mg ml^−1^ TOCL. Purified proteins were injected onto the columns at 0.5 ml min^−1^. The dn/dc values for LMNG-TOCL and DDM-TOCL were experimentally determined using the refractive index detector. Detergent-lipid solutions (5:1 detergent:lipid ratio for LMNG-TOCL and 10:1 detergent-to-lipid ratio for DDM-TOCL) with defined concentrations were prepared in 20 mM Hepes, pH 8.0 and 150 mM NaCl. Solutions were injected into the refractive index detector. All data were recorded and analysed with ASTRA 6.03 (Wyatt Technologies). Molecular weight calculations were performed using the protein-conjugate method (known as the three-detector method^29^) with the dn/dc value for protein of 0.185 ml g^−1^ and the measured dn/dc value for LMNG-TOCL of 0.1675, ml g^−1^ (citrin) and for DDM-TOCL of 0.1605, ml g^−1^ (carrier domain of citrin and Aac2p). The extinction coefficient *ε*_A280_ for citrin, the carrier domain of citrin and Aac2p were calculated from the amino-acid sequence using the ProtParam tool on the ExPaSy server^64^, to determine the contribution of the protein to the overall protein-detergent-lipid (PDL) complex. Molecular weight calculations for the N-terminal domain of aralar and the N- and C-terminal domain fusions of aralar and citrin were calculated using the standard two-detector method^29^.

### Probing the interaction of citrin N- and C-terminal domains

The fusion of the N- and C-terminal domains of citrin was purified as previously described with minor changes in the buffer at the size-exclusion step, as described below. The eluted sample was concentrated to a volume of 1 ml in a 30-kDa MWCO centrifugal concentrator and split into two samples labelled sample ‘C’ and sample ‘E’. Sample ‘C’ was supplemented with 5 mM CaCl_2_ and 5 mM DTT, whereas sample ‘E’ was supplemented with 10 mM EGTA, 10 mM EDTA and 5 mM DTT. Sample C was injected onto a Superdex 200 HiLoad 16/60 120 pg column at 1 ml min^−1^ with SEC buffer C (20 mM Hepes, pH 7.5, 150 mM NaCl, 5 mM CaCl_2_) and sample E was injected onto the same column with SEC buffer E (20 mM Hepes, pH 7.5, 150 mM NaCl, 10 mM EGTA, pH 7.5). Peak fractions were pooled, concentrated to 8.4 mg ml^−1^ and supplemented with 5 mM DTT. N- and C-terminal domains were cleaved by 150 μg MBP-TEV overnight. MBP-TEV was removed by amylose affinity chromatography. TEV-treated samples were further separated on a Superose 12 10/30 HR column (GE Healthcare) at 0.2 ml min^−1^ with the respective buffers for samples C and E on an ÄKTA micro. Peak fractions were run on 10% Tris-tricine gels.

## Additional information

**How to cite this article:** Thangaratnarajah, C. *et al.* Calcium-induced conformational changes of the regulatory domain of human mitochondrial aspartate/glutamate carriers. *Nat. Commun.* 5:5491 doi: 10.1038/ncomms6491 (2014).

**Accession codes**. The coordinates and structure factors of the N- and C-terminal fusion of citrin and the calcium-bound and calcium-free forms of the N-terminal domain of aralar have been deposited in the Protein Data Bank under accession codes 4P5W, 4P5X and 4P60, respectively.

## Supplementary Material

Supplementary Information Supplementary Figures 1-7, Supplementary Table 1, Supplementary Methods and Supplementary References

Supplementary Movie 1 Morph between the calcium-bound state of citrin and the calcium-free state of aralar (dimer created from chain B). Structures are shown as cartoons, and colored according to Fig. 1.

## References

[b1] LaNoue K. F. & Tischler M. E. Electrogenic characteristics of the mitochondrial glutamate-aspartate antiporter. J. Biol. Chem. 249, 7522–7528 (1974). 4436323

[b2] LaNoue K. F., Bryla J. & Bassett D. J. Energy-driven aspartate efflux from heart and liver mitochondria. J. Biol. Chem. 249, 7514–7521 (1974). 4436322

[b3] LaNoue K. F., Meijer A. J. & Brouwer A. Evidence for electrogenic aspartate transport in rat liver mitochondria. Arch. Biochem. Biophys. 161, 544–550 (1974). 4839046 10.1016/0003-9861(74)90337-3

[b4] Dierks T. & Krämer R. Asymmetric orientation of the reconstituted aspartate/glutamate carrier from mitochondria. Biochim. Biophys. Acta 937, 112–126 (1988). 3334841 10.1016/0005-2736(88)90233-7

[b5] Dierks T., Riemer E. & Krämer R. Reaction mechanism of the reconstituted aspartate/glutamate carrier from bovine heart mitochondria. Biochim. Biophys. Acta 943, 231–244 (1988). 2900025 10.1016/0005-2736(88)90555-x

[b6] Palmieri L. *et al.* Citrin and aralar1 are Ca(2+)-stimulated aspartate/glutamate transporters in mitochondria. EMBO J. 20, 5060–5069 (2001). 11566871 10.1093/emboj/20.18.5060 PMC125626

[b7] Azzi A., Chappell J. B. & Robinson B. H. Penetration of the mitochondrial membrane by glutamate and aspartate. Biochem. Biophys. Res. Commun. 29, 148–152 (1967). 6055180 10.1016/0006-291x(67)90556-6

[b8] Del Arco A. & Satrústegui J. Molecular cloning of Aralar, a new member of the mitochondrial carrier superfamily that binds calcium and is present in human muscle and brain. J. Biol. Chem. 273, 23327–23334 (1998). 9722566 10.1074/jbc.273.36.23327

[b9] Kobayashi K. *et al.* The gene mutated in adult-onset type II citrullinaemia encodes a putative mitochondrial carrier protein. Nat. Genet. 22, 159–163 (1999). 10369257 10.1038/9667

[b10] Del Arco A., Agudo M. & Satrústegui J. Characterization of a second member of the subfamily of calcium-binding mitochondrial carriers expressed in human non-excitable tissues. Biochem. J. 345, (Pt 3): 725–732 (2000). 10642534 10.1042/0264-6021:3450725 PMC1220810

[b11] LaNoue K. F. & Schoolwerth A. C. Metabolite transport in mitochondria. Annu. Rev. Biochem. 48, 871–922 (1979). 38739 10.1146/annurev.bi.48.070179.004255

[b12] Borst P. Hydrogen Transport And Transport Metabolites. inFunktionelle und Morphologische Organisation der Zelle ed. Karson P. 137–162Springer Verlag (1963).

[b13] Williamson J. R. inGluconeogenesis: Its Regulation in Mammalian Species eds Hanson R., Mehlman M. 165–220John Wiley (1976).

[b14] Meijer A. J. *et al.* Interrelationships between gluconeogenesis and ureogenesis in isolated hepatocytes. J. Biol. Chem. 253, 2308–2320 (1978). 632271

[b15] Jalil M. A. *et al.* Reduced N-acetylaspartate levels in mice lacking aralar, a brain- and muscle-type mitochondrial aspartate-glutamate carrier. J. Biol. Chem. 280, 31333–31339 (2005). 15987682 10.1074/jbc.M505286200

[b16] Wibom R. *et al.* AGC1 deficiency associated with global cerebral hypomyelination. New Engl. J. Med. 361, 489–495 (2009). 19641205 10.1056/NEJMoa0900591

[b17] Contreras L. *et al.* Ca^2+^ Activation kinetics of the two aspartate-glutamate mitochondrial carriers, aralar and citrin: role in the heart malate-aspartate NADH shuttle. J. Biol. Chem. 282, 7098–7106 (2007). 17213189 10.1074/jbc.M610491200

[b18] Lasorsa F. M. *et al.* Recombinant expression of the Ca^2+^-sensitive aspartate/glutamate carrier increases mitochondrial ATP production in agonist-stimulated chinese hamster ovary cells. J. Biol. Chem. 278, 38686–38692 (2003). 12851387 10.1074/jbc.M304988200

[b19] Pardo B. *et al.* Essential role of aralar in the transduction of small Ca^2+^ signals to neuronal mitochondria. J. Biol. Chem. 281, 1039–1047 (2006). 16269409 10.1074/jbc.M507270200

[b20] Mármol P. *et al.* Requirement for aralar and its Ca^2+^-binding sites in Ca2+ signal transduction in mitochondria from INS-1 clonal beta-cells. J. Biol. Chem. 284, 515–524 (2009). 18996845 10.1074/jbc.M806729200

[b21] Pebay-Peyroula E. *et al.* Structure of mitochondrial ADP/ATP carrier in complex with carboxyatractyloside. Nature 426, 39–44 (2003). 14603310 10.1038/nature02056

[b22] Ruprecht J. J. *et al.* Structures of yeast mitochondrial ADP/ATP carriers support a domain-based alternating-access transport mechanism. Proc. Natl Acad. Sci. USA 111, E426–E434 (2014). 24474793 10.1073/pnas.1320692111 PMC3910652

[b23] Kunji E. R. & Harding M. Projection structure of the atractyloside-inhibited mitochondrial ADP/ATP carrier of *Saccharomyces cerevisiae*. J. Biol. Chem. 278, 36985–36988 (2003). 12893834 10.1074/jbc.C300304200

[b24] Bamber L., Harding M., Butler P. J. & Kunji E. R. Yeast mitochondrial ADP/ATP carriers are monomeric in detergents. Proc. Natl Acad. Sci. USA 103, 16224–16229 (2006). 17056710 10.1073/pnas.0607640103 PMC1618811

[b25] Bamber L., Harding M., Monne M., Slotboom D. J. & Kunji E. R. The yeast mitochondrial ADP/ATP carrier functions as a monomer in mitochondrial membranes. Proc. Natl Acad. Sci. USA 104, 10830–10834 (2007). 17566106 10.1073/pnas.0703969104 PMC1891095

[b26] Nury H. *et al.* Mitochondrial bovine ADP/ATP carrier in detergent is predominantly monomeric but also forms multimeric species. Biochemistry 47, 12319–12331 (2008). 18980386 10.1021/bi801053m

[b27] Kunji E. R. & Crichton P. G. Mitochondrial carriers function as monomers. Biochim. Biophys. Acta 1797, 817–831 (2010). 20362544 10.1016/j.bbabio.2010.03.023

[b28] Chae P. S. *et al.* Maltose-neopentyl glycol (MNG) amphiphiles for solubilization, stabilization and crystallization of membrane proteins. Nat. Methods 7, 1003–1008 (2010). 21037590 10.1038/nmeth.1526 PMC3063152

[b29] Slotboom D. J., Duurkens R. H., Olieman K. & Erkens G. B. Static light scattering to characterize membrane proteins in detergent solution. Methods 46, 73–82 (2008). 18625320 10.1016/j.ymeth.2008.06.012

[b30] Robinson A. J., Overy C. & Kunji E. R. The mechanism of transport by mitochondrial carriers based on analysis of symmetry. Proc. Natl Acad. Sci. USA 105, 17766–17771 (2008). 19001266 10.1073/pnas.0809580105 PMC2582046

[b31] Miroux B., Frossard V., Raimbault S., Ricquier D. & Bouillaud F. The topology of the brown adipose tissue mitochondrial uncoupling protein determined with antibodies against its antigenic sites revealed by a library of fusion proteins. EMBO J. 12, 3739–3745 (1993). 7691596 10.1002/j.1460-2075.1993.tb06051.x PMC413655

[b32] Capobianco L., Brandolin G. & Palmieri F. Transmembrane topography of the mitochondrial phosphate carrier explored by peptide-specific antibodies and enzymatic digestion. Biochemistry 30, 4963–4969 (1991). 2036364 10.1021/bi00234a018

[b33] Yap K. L., Ames J. B., Swindells M. B. & Ikura M. Diversity of conformational states and changes within the EF-hand protein superfamily. Proteins Struct. Funct. Genet. 37, 499–507 (1999). 10591109 10.1002/(sici)1097-0134(19991115)37:3<499::aid-prot17>3.0.co;2-y

[b34] Gifford J. L., Walsh M. P. & Vogel H. J. Structures and metal-ion-binding properties of the Ca^2+^-binding helix-loop-helix EF-hand motifs. Biochem. J. 405, 199–221 (2007). 17590154 10.1042/BJ20070255

[b35] Kirberger M. *et al.* Integration of Diverse Research Methods to Analyze and Engineer Ca-Binding Proteins: From Prediction to Production. Curr. Bioinform. 5, 68–80 (2010). 20802832 10.2174/157489310790596358 PMC2927018

[b36] Kretsinger R. H., Rudnick S. E. & Weissman L. J. Crystal structure of calmodulin. J. Inorg. Biochem. 28, 289–302 (1986). 3806094 10.1016/0162-0134(86)80093-9

[b37] Meador W. E., Means A. R. & Quiocho F. A. Target enzyme recognition by calmodulin: 2.4 A structure of a calmodulin-peptide complex. Science 257, 1251–1255 (1992). 1519061 10.1126/science.1519061

[b38] Réty S. *et al.* The crystal structure of a complex of p11 with the annexin II N-terminal peptide. Nat. Struct. Biol. 6, 89–95 (1999). 9886297 10.1038/4965

[b39] Réty S. *et al.* Structural basis of the Ca(2+)-dependent association between S100C (S100A11) and its target, the N-terminal part of annexin I. Structure 8, 175–184 (2000). 10673436 10.1016/s0969-2126(00)00093-9

[b40] Song Y. Z. *et al.* *SLC25A13* gene analysis in citrin deficiency: sixteen novel mutations in East Asian patients, and the mutation distribution in a large pediatric cohort in China. PLoS ONE 8, e74544 (2013). 24069319 10.1371/journal.pone.0074544 PMC3777997

[b41] Dimmock D. *et al.* Citrin deficiency, a perplexing global disorder. Mol. Genet. Metab. 96, 44–49 (2009). 19036621 10.1016/j.ymgme.2008.10.007

[b42] Woo H. I., Park H. D. & Lee Y. W. Molecular genetics of citrullinemia types I and II. Clin. Chim. Acta 431C, 1–8 (2014). 10.1016/j.cca.2014.01.032 24508627

[b43] Saheki T., Inoue K., Tushima A., Mutoh K. & Kobayashi K. Citrin deficiency and current treatment concepts. Mol. Genet. Metab. 100, (Suppl 1): S59–S64 (2010). 20233664 10.1016/j.ymgme.2010.02.014

[b44] Saheki T. *et al.* Pathogenesis and pathophysiology of citrin (a mitochondrial aspartate glutamate carrier) deficiency. Metab. Brain Dis. 17, 335–346 (2002). 12602510 10.1023/a:1021961919148

[b45] Liu G. *et al.* A novel mutation of the *SLC25A13* gene in a Chinese patient with citrin deficiency detected by target next-generation sequencing. Gene 533, 547–553 (2014). 24161253 10.1016/j.gene.2013.10.021

[b46] Zhang Z. H. *et al.* Clinical, molecular and functional investigation on an infant with neonatal intrahepatic cholestasis caused by citrin deficiency (NICCD). PLoS ONE 9, e89267 (2014). 24586645 10.1371/journal.pone.0089267 PMC3931723

[b47] Fu H. Y. *et al.* The mutation spectrum of the *SLC25A13* gene in Chinese infants with intrahepatic cholestasis and aminoacidemia. J. Gastroenterol. 46, 510–518 (2011). 20927635 10.1007/s00535-010-0329-y

[b48] Kunji E. R., Slotboom D. J. & Poolman B. *Lactococcus lactis* as host for overproduction of functional membrane proteins. Biochim. Biophys. Acta 1610, 97–108 (2003). 12586384 10.1016/s0005-2736(02)00712-5

[b49] Nurizzo D. *et al.* The ID23-1 structural biology beamline at the ESRF. J. Synchrotron. Radiat. 13, 227–238 (2006). 16645249 10.1107/S0909049506004341

[b50] Flot D. *et al.* The ID23-2 structural biology microfocus beamline at the ESRF. J. Synchrotron. Radiat. 17, 107–118 (2010). 20029119 10.1107/S0909049509041168 PMC3025444

[b51] Kabsch W. XDS. Acta Crystallogr. D Biol. Crystallogr. 66, 125–132 (2010). 20124692 10.1107/S0907444909047337 PMC2815665

[b52] Kabsch W. Integration, scaling, space-group assignment and post-refinement. Acta Crystallogr. D Biol. Crystallogr. 66, 133–144 (2010). 20124693 10.1107/S0907444909047374 PMC2815666

[b53] Evans P. R. An introduction to data reduction: space-group determination, scaling and intensity statistics. Acta Crystallogr. D Biol. Crystallogr. 67, 282–292 (2011). 21460446 10.1107/S090744491003982X PMC3069743

[b54] Winn M. D. *et al.* Overview of the CCP4 suite and current developments. Acta Crystallogr. D Biol. Crystallogr. 67, 235–242 (2011). 21460441 10.1107/S0907444910045749 PMC3069738

[b55] Schneider T. R. & Sheldrick G. M. Substructure solution with SHELXD. Acta Crystallogr. D Biol. Crystallogr. 58, 1772–1779 (2002). 12351820 10.1107/s0907444902011678

[b56] Vonrhein C., Blanc E., Roversi P. & Bricogne G. Automated structure solution with autoSHARP. Methods Mol. Biol. 364, 215–230 (2007). 17172768 10.1385/1-59745-266-1:215

[b57] Cowtan K. The Buccaneer software for automated model building. 1. Tracing protein chains. Acta Crystallogr. D Biol. Crystallogr. 62, 1002–1011 (2006). 16929101 10.1107/S0907444906022116

[b58] Cowtan K. Fitting molecular fragments into electron density. Acta Crystallogr. D Biol. Crystallogr. 64, 83–89 (2008). 18094471 10.1107/S0907444907033938 PMC2394793

[b59] Emsley P., Lohkamp B., Scott W. G. & Cowtan K. Features and development of Coot. Acta Crystallogr. D Biol. Crystallogr. 66, 486–501 (2010). 20383002 10.1107/S0907444910007493 PMC2852313

[b60] Murshudov G. N. *et al.* REFMAC5 for the refinement of macromolecular crystal structures. Acta Crystallogr. D Biol. Crystallogr. 67, 355–367 (2011). 21460454 10.1107/S0907444911001314 PMC3069751

[b61] Afonine P. V. *et al.* Towards automated crystallographic structure refinement with phenix.refine. Acta Crystallogr. D Biol. Crystallogr. 68, 352–367 (2012). 22505256 10.1107/S0907444912001308 PMC3322595

[b62] Chen V. B. *et al.* MolProbity: all-atom structure validation for macromolecular crystallography. Acta. Crystallogr. D. Biol. Crystallogr. 66, 12–21 (2010). 20057044 10.1107/S0907444909042073 PMC2803126

[b63] McCoy A. J. *et al.* Phaser crystallographic software. J. Appl. Crystallogr. 40, 658–674 (2007). 19461840 10.1107/S0021889807021206 PMC2483472

[b64] Gasteiger E. *et al.* inThe Proteomics Protocols Handbook ed. Walker J. M. 571–607Humana Press (2005).

[b65] Bunkoczi G. & Read R. J. Improvement of molecular-replacement models with Sculptor. Acta Crystallogr. D Biol. Crystallogr. 67, 303–312 (2011). 21460448 10.1107/S0907444910051218 PMC3069745

[b66] Terwilliger T. C. *et al.* Iterative model building, structure refinement and density modification with the PHENIX AutoBuild wizard. Acta Crystallogr. D Biol. Crystallogr. 64, 61–69 (2008). 18094468 10.1107/S090744490705024X PMC2394820

[b67] Terwilliger T. C. Maximum-likelihood density modification. Acta Crystallogr. D Biol. Crystallogr 56, 965–972 (2000). 10944333 10.1107/S0907444900005072 PMC2792768

[b68] Pettersen E. F. *et al.* UCSF Chimera--a visualization system for exploratory research and analysis. J. Comput. Chem. 25, 1605–1612 (2004). 15264254 10.1002/jcc.20084

[b69] Krissinel E. & Henrick K. Inference of macromolecular assemblies from crystalline state. J. Mol. Biol. 372, 774–797 (2007). 17681537 10.1016/j.jmb.2007.05.022

[b70] Hayward S. & Berendsen H. J. Systematic analysis of domain motions in proteins from conformational change: new results on citrate synthase and T4 lysozyme. Proteins 30, 144–154 (1998). 9489922

