# Supplementary material for: Calcium-induced conformational changes of the regulatory domain of human mitochondrial aspartate/glutamate carriers
Source: Nat Commun. 2014 Nov 20;5:5491. doi: 10.1038/ncomms6491 (PMC4250520; doi:10.1038/ncomms6491)
Supplement: Supplementary Information — Supplementary Figures 1-7, Supplementary Table 1, Supplementary Methods and Supplementary References [file ncomms6491-s1.pdf]

Supplementary information

Calcium-induced conformational changes of the regulatory domain of human mitochondrial aspartate/glutamate carriers

Chancievan Thangaratnarajah[1], Jonathan J. Ruprecht[1], and Edmund R.S. Kunji*

The Medical Research Council, Mitochondrial Biology Unit, Wellcome Trust/MRC Building, Hills Road, Cambridge, CB2 0XY, United Kingdom

[1]These authors contributed equally.
*To whom correspondence should be addressed. E-mail: ek@mrc-mbu.cam.ac.uk, Tel: +44 (0)1223 252850

Supplementary information contains:

Supplementary Figures 1-7

Supplementary Table 1

Supplementary Methods

Supplementary References

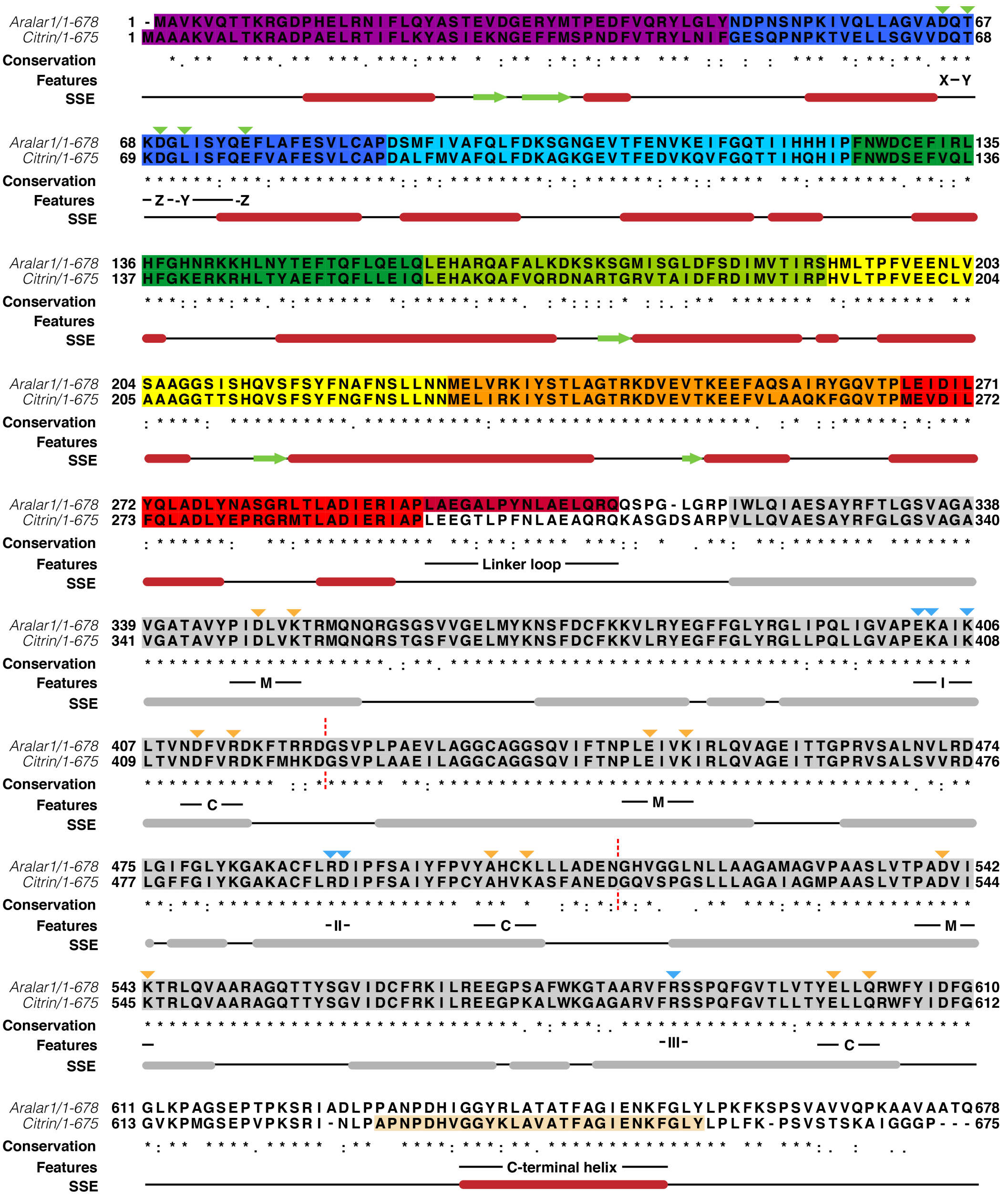

Supplementary Figure 1 Amino acid sequence alignment of aralar and citrin. Pairwise alignment of aralar (Uniprot: O75746) and citrin (Q9UJS0) using Clustal O. Sequence conservation is indicated with an asterix for identical residues, a colon for conserved substitutions, a period for semi-conserved substitutions, and a gap for non-conserved residues. EF-hand regions are colored as shown in Figure 1a and 2. The linker loop, only resolved in the N-terminal domain of aralar in the calcium-free state, is colored dark red. The C-terminal helix, only resolved in N- and C-terminal domain fusion of citrin, is colored wheat. The carrier domain is shown in grey. Secondary structural elements (SSE) are shown as

follows: the α-helices and β-sheets of the regulatory domain are shown as red bars and green arrows, respectively, and the α-helices of the carrier domain as grey bars. Green arrowheads indicate the residues that are involved in the coordination of calcium, and X, Y, Z, -Y and -Z indicate the axial positions in space. Black lines label the positions of the linker loop and the C-terminal helix. The charged residues of the Px[DE]xx[KR] motif, forming the matrix salt-bridge network (M), and the [YF][DE]xx[KR] motif, forming the cytoplasmic salt-bridge network (C), are indicated by orange arrowheads. The contact points of the substrate-binding site are indicated with blue arrowheads and are labelled with roman numerals according to Robinson et al. (2008) . Red dotted vertical lines show the borders of the three homologous repeats in the carrier domain.

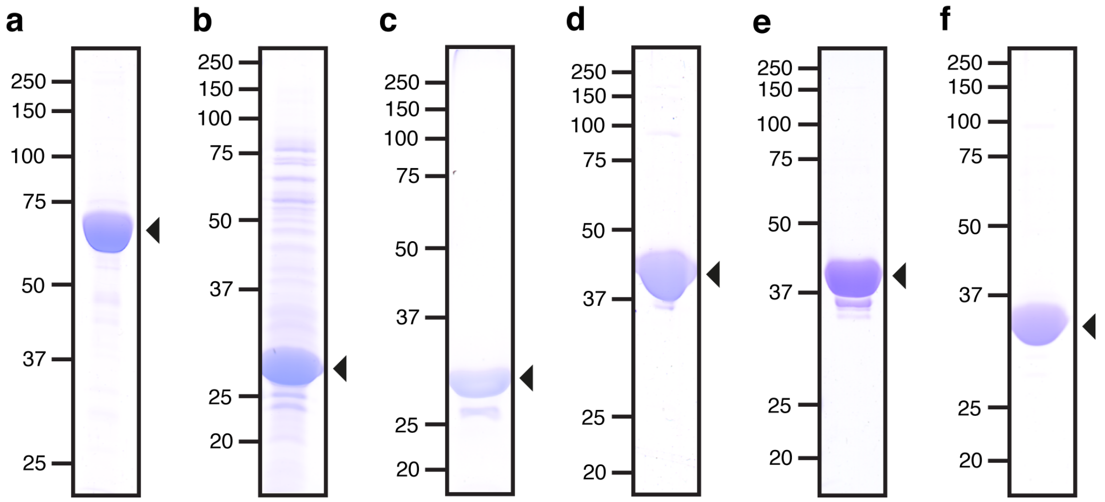

Supplementary Figure 2 Purification of domains of citrin and aralar for SEC-MALLS analysis. SDS-PAGE analysis of purifications of (**a**) citrin in lauryl maltose neopentyl glycol, (**b**) carrier domain of citrin in dodecylmaltoside, (**c**) CATR-inhibited Aac2p in dodecylmaltoside, (**d**) N- and C-terminal domain fusion of citrin, (**e**) N- and C-terminal domain fusion of aralar, containing a R339G mutation to remove a non-canonical factor Xa cleavage site, and (**f**) N-terminal domain of aralar. All samples (10 µg) were analyzed on 12 % SDS-PAGE gels except for citrin, which was analysed on a 10 % SDS-PAGE gel.

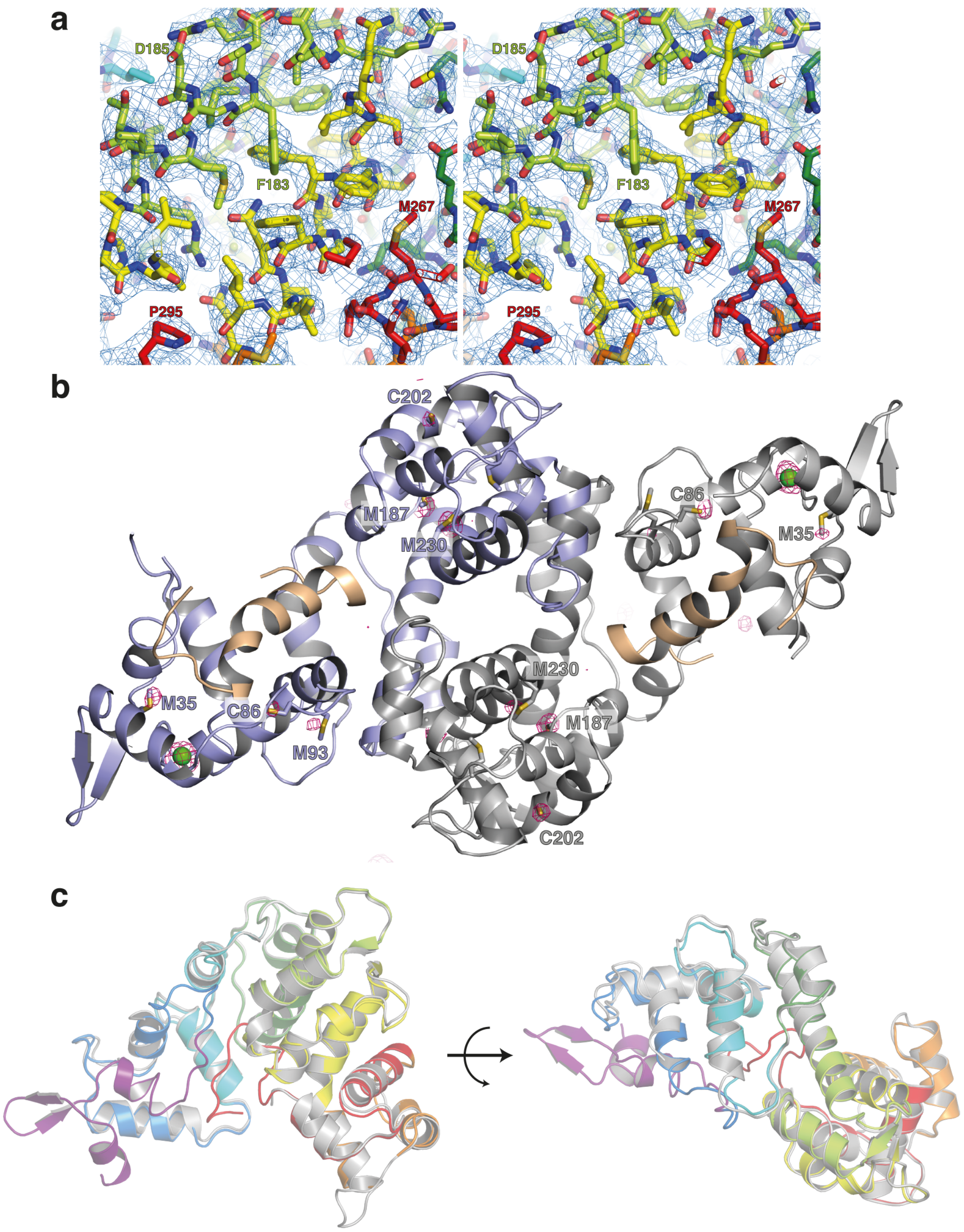

Supplementary Figure 3 Crystallographic analysis of citrin and aralar. (**a**) Stereo view of the experimental MIRAS electron density map of citrin after density modification. The map is shown as a blue mesh, contoured at 1σ. The final refined model is shown in stick representation, colored according to **Fig. 1a**. Labelled residues are from chain B. (**b**) Anomalous difference Fourier map calculated to 3.5 Å resolution from data collected at low energy (6500 eV X-rays). The map is shown as a red mesh, contoured at 4σ. Peaks are

observed at the position of the Ca^{2+} ions (green spheres) and near the sulfur atoms of cysteine and methionine residues. Chains A and B of citrin are colored light blue and grey, respectively, and the C-terminal helix is colored in wheat, and they are viewed from the mitochondrial matrix. (**c**) Comparison of chains A and B of the aralar calcium-free state structure. Both chains are shown in cartoon representation, with chain A in grey and chain B colored as in Figure 1a and 2. The chains were aligned by superimposing EF hands 4-8. Chain A lacks density for EF hand 1, but EF hands 2-3 are in the same conformation as found in chain B.

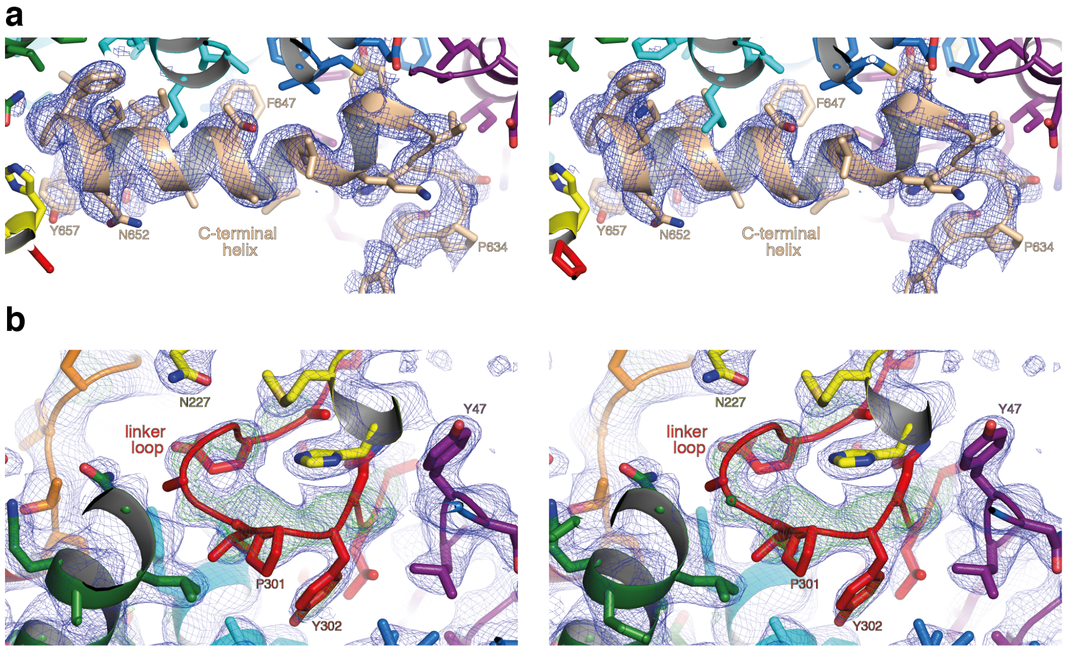

Supplementary Figure 4 Stereo views of the density maps of the C-terminal helix and linker loop. (**a**) Experimental electron density map of citrin calculated using density-modified MIRAS phases. The electron density map is contoured at 1σ, and is shown as a blue mesh within 2.5 Å of the atoms of the C-terminal region. (**b**) Simulated-annealing OMIT map of the linker loop region of the calcium-free state of aralar. The map was calculated by omitting residues of the linker loop (Ala296-Gln308) before refinement in phenix.refine[2] (3 macrocycles of coordinate, B-factor and torsion angle simulated annealing refinement with a starting temperature of 2500 K). The resulting $2mF_o$-DF_c map is shown as a blue mesh, contoured at 0.8σ, and the mF_o-DF_c map is shown as a green mesh at 3.0σ. Chain A is depicted in (**a**) and chain B in (**b**), colored according to the scheme in **Fig. 3**.

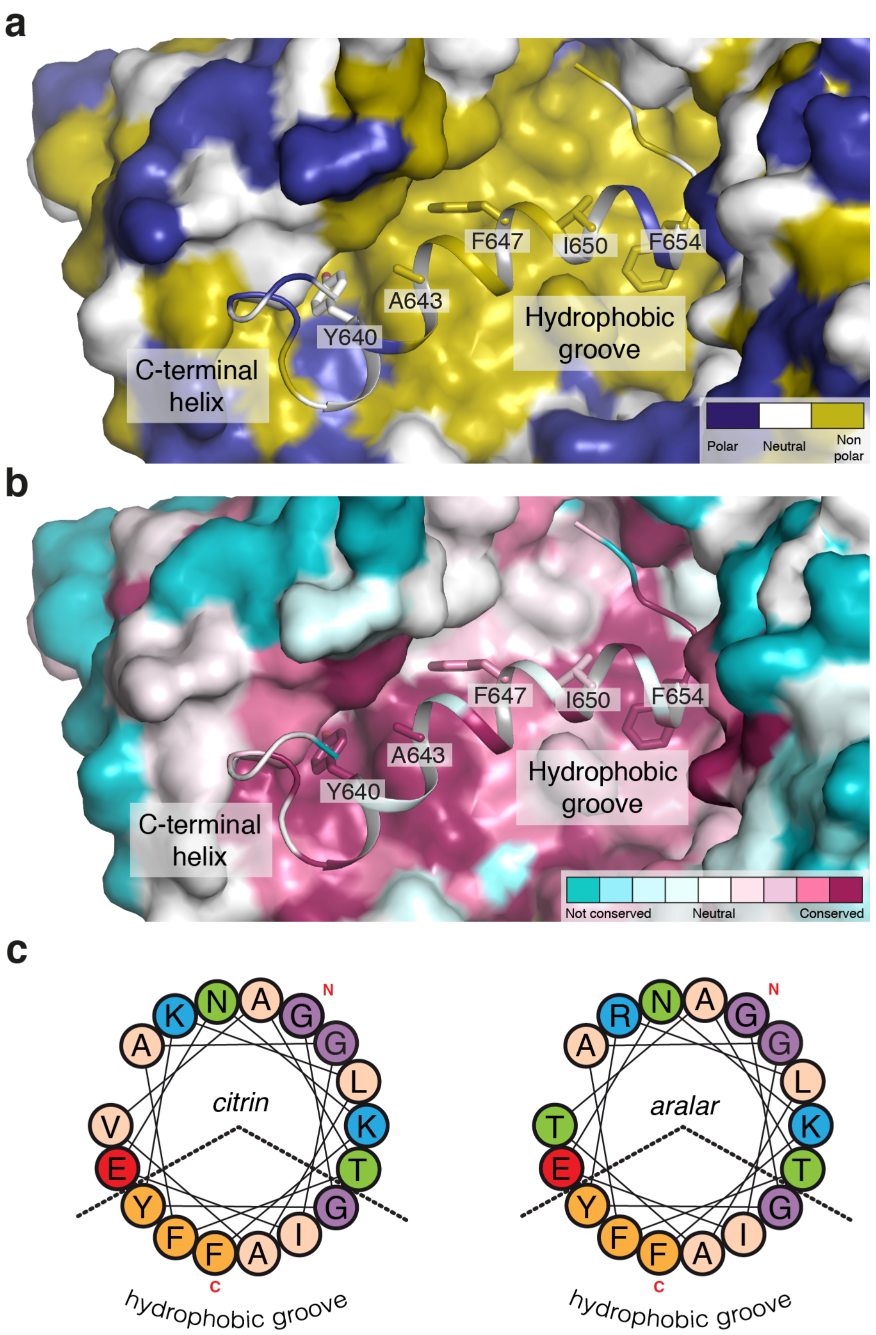

Supplementary Figure 5 Binding environment and conservation of the amphipathic helix of the C-terminal domain. (**a**) The N-terminal domain is shown in surface representation with the C-terminal domain bound to the hydrophobic groove in cartoon representation. Both are colored with a coloring scheme displaying hydrophobicity according to Kyte and Doolittle (1982) . Polar residues (Arg, Lys, Asn, Asp, Gln, Glu, His) are colored in dark blue, neutral residues (Pro, Tyr, Ser, Trp, Thr, Gly) in white and non-polar residues (Ala, Met, Cys, Phe, Leu, Val, Ile) in olive. (**b**) The same view as described in (**a**), but displaying sequence conservation. The sequence conservation scores were calculated using the Consurf server[4],

with a multiple-sequence alignment generated from sequences encoding metazoan orthologs of the human aspartate/glutamate carriers. Labeled residues indicate hydrophobic residues of the C-terminal domain facing the hydrophobic groove. (**c**) Helical wheel plot for residues 639 – 654 of citrin and residues 638 – 653 of aralar, corresponding to the α-helix of the C-terminal domain. The helical wheel plots were generated using HELIQUEST[5]. Residues are colored according to the Zappo color scheme and the N- and C-termini of the C-terminal helix are indicated in red. Dotted black lines indicate the boundaries of residues facing the hydrophobic groove in the N-terminal domain.

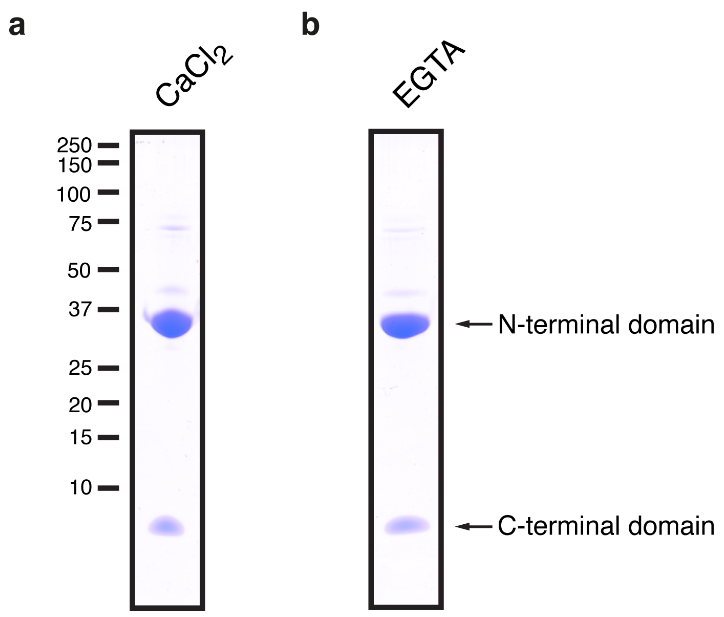

Supplementary Figure 6 Interaction of the N-terminal and C-terminal domain. An engineered TEV cleavage site in the linker between the N- and C-terminal domains was hydrolysed by TEV protease in (**a**) the presence of calcium (5 mM) or (**b**) in the presence of EGTA (10 mM) to remove calcium, and the cleavage products were separated on a Superose 12 size-exclusion column. The peak fraction was run on a 10 % Tris-tricine gel.

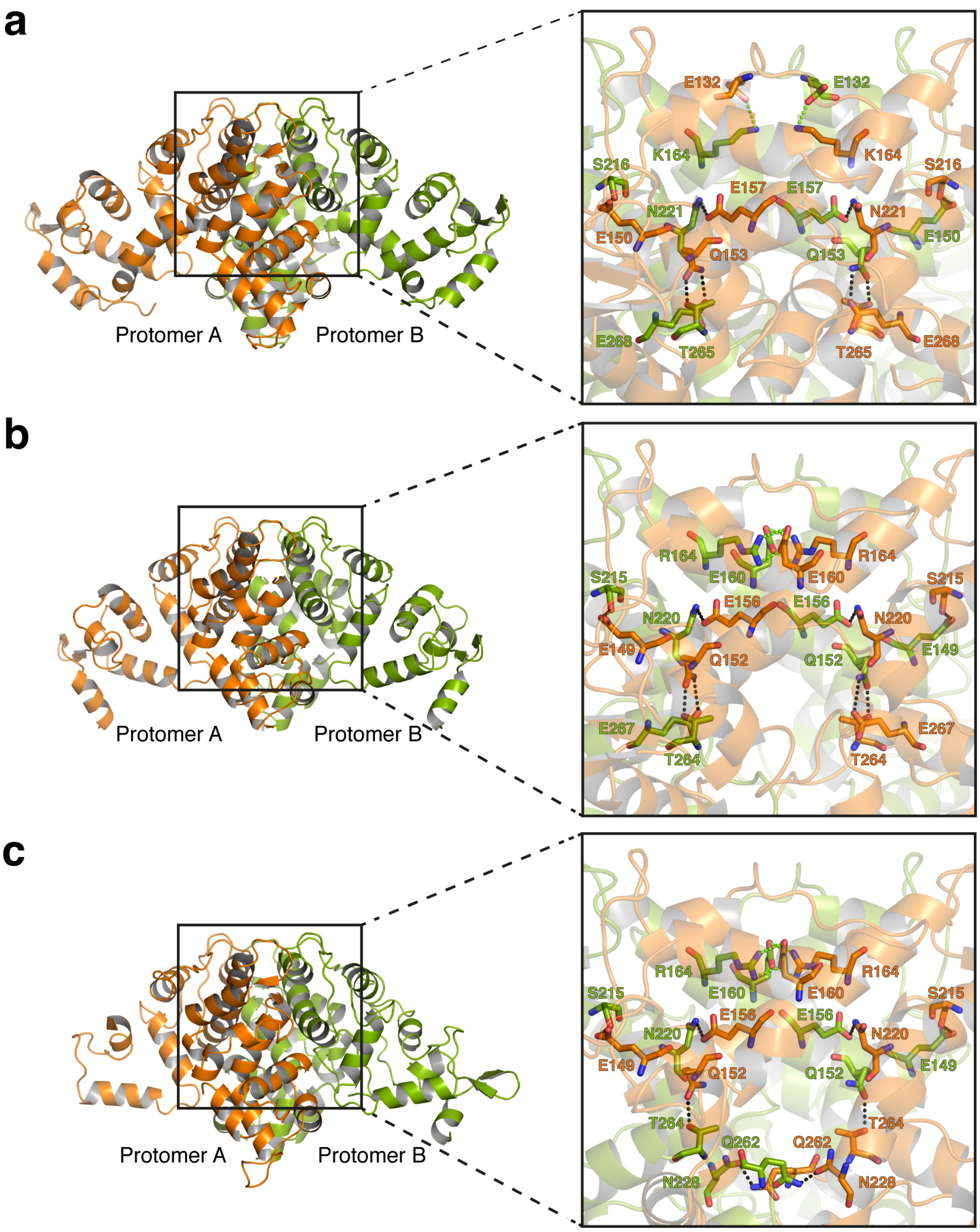

Supplementary Figure 7 Dimerization interface of the N-terminal domain. Residues forming side chain-mediated hydrogen bonds and salt bridges in the dimerization interface of (**a**) N- and C-terminal domain fusion of citrin, (**b**) calcium-bound and (**c**) calcium-free states of the N-terminal domain of aralar. Protomers A and B are shown in orange and green, respectively. The areas of the close-ups are indicated by black squares in the overall structures. Hydrogen bonds and salt bridge interactions are shown as dotted lines in black and green, respectively. The residues involved in side chain interactions are shown in stick representation.

Supplementary Table 1: Data collection, phasing and refinement statistics for citrin (MIRAS)

	Native-1[a]	EMTS-1[a]	EMTS-2[a]	K_2PtCl_4[a]	$KAu(CN)_2$-1[a]	$KAu(CN)_2$-2[a]	Native-2[a] (anomalous)
**Data collection**							
Space group	$P2_12_12_1$	$P2_12_12_1$	$P2_12_12_1$	$P2_12_12_1$	$P2_12_12_1$	$P2_12_12_1$	$P2_12_12_1$
Cell dimensions							
a, *b*, *c* (Å)	68.15, 106.2, 117.9	67.96, 106.4, 117.5	68.15, 106.6, 117.8	67.86, 105.7, 117.5	68.02, 105.3, 117.7	67.85, 105.3, 117.4	67.95, 106.3, 118.0
α, β, γ (°)	90.0, 90.0, 90.0	90.0, 90.0, 90.0	90.0, 90.0, 90.0	90.0, 90.0, 90.0	90.0, 90.0, 90.0	90.0, 90.0, 90.0	90.0, 90.0, 90.0
Wavelength (Å)	0.8726	1.0056	1.00238	1.06433	1.03285	1.03285	1.9075
Resolution (Å)[b]	53.08-2.40 (2.49-2.40)	58.83-2.89 (3.06-2.89)	59.0-3.30 (3.56-3.30)	58.76-3.40 (3.67-3.40)	58.89-3.10 (3.31-3.10)	58.75-2.66 (2.79-2.66)	29.49-2.98 (3.16-2.98)
R_{merge} (all I+ and I-)	0.074 (0.685)	0.094 (0.649)	0.118 (0.519)	0.165 (0.538)	0.096 (0.444)	0.122 (0.916)	0.101 (0.646)
$<<I>/\sigma(<I>)>$	13.2 (2.0)	11.5 (2.0)	14.9 (4.0)	12.4 (4.1)	16.0 (4.4)	12.1 (1.8)	17.1 (3.4)
Completeness (%)	99.9 (99.9)	95.4 (96.6)	99.9 (100.0)	99.9 (99.9)	99.9 (100.0)	99.7 (98.1)	99.1 (95.0)
Redundancy	3.7 (3.8)	3.1 (3.1)	6.4 (6.8)	6.3 (6.6)	6.4 (6.4)	6.4 (5.3)	9.0 (8.3)
Anomalous completeness (%)		82.0 (82.6)	99.5 (100.0)	99.5 (99.4)	99.6 (99.6)	99.1 (95.3)	98.8 (93.2)
Anomalous Redundancy		1.5 (1.4)	3.4 (3.5)	3.3 (3.3)	3.3 (3.2)	3.2 (2.6)	4.7 (4.2)
**Refinement**							
Resolution (Å)	53.08-2.40						
No. reflections	34085						
R_{work} / R_{free}	19.33/22.69						
No. atoms	5087						
Protein	4937						
Ligand/ion	2						
Water	148						
B-factors	59.20						
Protein	59.50						
Ligand/ion	79.30						
Water	49.10						
R.m.s deviations							
Bond lengths (Å)	0.005						
Bond angles (°)	0.79						

[a] Each dataset was collected from a single crystal.

[b] Values in parentheses are for highest-resolution shell.

Data collection and refinement statistics for aralar (molecular replacement)

	Aralar (calcium-bound)[a]	Aralar (calcium-free)[a]
**Data collection**		
Space group	$P3_121$	$P6_322$
Cell dimensions		
a, *b*, *c* (Å)	54.5, 54.5, 168.2	103.5, 103.5, 185.6
α, β, γ (°)	90, 90, 20	90, 90, 120
Resolution (Å)[b]	28.03-2.26 (2.34-2.26)	29.50-2.40 (2.49-2.40)
R_{merge}	0.044 (0.859)	0.077 (1.242)
$\langle\langle I\rangle/\sigma\langle I\rangle\rangle$	21.3 (2.2)	19.0 (1.7)
Completeness (%)	99.8 (99.8)	99.7 (98.3)
Redundancy	6.4 (6.3)	9.2 (7.8)
**Refinement**		
Resolution (Å)	28.03-2.26	29.50-2.40
No. reflections	14197	23702
R_{work} / R_{free}	23.12/27.41	24.52/29.66
No. atoms	2141	4102
Protein	2117	4080
Ligand/ion	1	1
Water	23	21
B-factors	81.10	78.40
Protein	81.30	78.50
Ligand/ion	52.80	61.70
Water	60.10	59.60
R.m.s. deviations		
Bond lengths (Å)	0.003	0.003
Bond angles (°)	0.53	0.65

[a] Each dataset was collected from a single crystal.
[b] Values in parentheses are for highest-resolution shell.

Supplementary Methods

Expression and purification of citrin

The gene encoding full-length citrin (Uniprot: Q9UJS0) with an N-terminal octa-histidine tag and Factor Xa-cleavage site was codon-optimised for expression in *Saccharomyces cerevisae* by GenScript. Subsequently, the Factor Xa cleavage site was replaced by a TEV-cleavage site by overlap extension PCR. The resulting gene was cloned into the yeast expression vector pYES3/CT (Invitrogen). The expression vectors were transformed into *Saccharomyces cerevisiae* strain W303-1B by the LiAC/SS carrier/PEG method[6]. Transformants were selected on SC-Trp + 2% (w/v) glucose agar plates and cultures were stored at -80°C.

To isolate mitochondria a 10 L starter culture was grown in SC-Trp + 2% (w/v) glucose at 30°C with shaking at 225 rpm overnight and used to inoculate a bioreactor with 100 L of YPG medium containing 0.1% (w/v) glucose. Expression of citrin was induced after 24 hours with 0.4% (w/v) galactose and cells were harvested after 4 hours by centrifugation. Cell pellets were washed with MilliQ water and stored at -80°C until further use. Cells were homogenised in 2 L breaking buffer (0.65 M sorbitol, 0.1 M Tris, pH 8.0, 5 mM EDTA, pH 8.0, 5 mM aminocaproic acid, 5 mM benzamidine) per kg of cells and supplemented with 1 mM PMSF. The cell suspension was passed through a bead mill (Dyno-Mill, Willy A. Bachofen AG) with 0.5–0.75 mm beads at a constant current of 4.5 A and a constant flow-rate of 3 litre per hour by using a peristaltic pump (Watson-Marlow 520S). Two low-speed centrifugation steps at 5,000g for 30 and 20 min, respectively, were used to remove unbroken cells and cell debris. Mitochondria were isolated by high-speed centrifugation at 25,500g for 1 hour. Following a wash step in wash buffer (same as breaking buffer, but without EDTA) crude mitochondria were resuspended in storage buffer (20 mM Hepes, pH 8.0, 10% (v/v) glycerol) and flash-frozen in liquid nitrogen in 250 mg aliquots. All steps were carried out at 4°C. One kg of yeast cells yielded approximately 6–8 g of mitochondria.

For the purification of citrin, mitochondria were buffered in solubilisation buffer (20 mM Hepes, pH 8.0, 150 mM NaCl, 20 mM imidazole, pH 8.0) with 8 EDTA-free protease inhibitor tablets (Roche) and solubilised with 1% (w/v) lauryl maltose neopentyl glycol (LMNG, Affimetrix). The solubilisate was clarified by ultracentrifugation at 205,000g for 1 hour and subsequently loaded onto a 1 ml Nickel-Sepharose High Performance column at 1 ml min^{-1} using an ÄKTA prime FPLC system. Unbound and non-specifically bound proteins were removed with 50 ml buffer A (20 mM Hepes, pH 8.0, 300 mM NaCl, 60 mM imidazole, pH 8.0, 0.05% (w/v) LMNG, 0.1 mg ml^{-1} 1,1',2,2'-tetraoleoyl-cardiolipin (TOCL, Avanti)) and 20 ml buffer B (20 mM Hepes, pH 8.0, 150 mM NaCl, 0.01% (w/v) LMNG, 0.02 mg ml^{-1} TOCL). Citrin was recovered by overnight on-column digestion with 540 μg of maltose-binding protein tagged TEV protease in the presence of 60 mM imidazole and 1 mM DTT. The mobile phase with untagged citrin was separated from the resin with spin columns (Proteus Midi Spin column, Generon) at 500g for 5 min. An additional 1.5 ml of buffer B supplemented with 60 mM imidazole was passed through the dry resin to recover residual citrin. Purified citrin was concentrated with a 100 kDa MWCO centrifugal concentrator (Sartorius, Germany) at 3,000g and injected onto a Superose 6 10/300 GL column equilibrated with SEC buffer (20 mM Hepes, pH 8.0, 150 mM NaCl, 0.002% (w/v) LMNG, 0.004 mg ml^{-1} TOCL) on an ÄKTA micro (GE Healthcare). Peak fractions were pooled and concentrated to 10 mg ml^{-1} with a 100 kDa MWCO centrifugal concentrator. Purified sample was flash-frozen and stored in liquid nitrogen.

Expression and purification of the carrier domain of citrin

The gene of the carrier domain of citrin (residues 320-615) was amplified from the gene of the full-length citrin and a sequence encoding an N-terminal octahistidine affinity tag and Factor Xa-cleavage site was introduced by PCR. The resulting gene was cloned into the yeast expression vector pYES3/CT. The expression vector was transformed into *Saccharomyces cerevisiae* strain W303-1B by the LiAC/SS carrier/PEG method[6]. Transformants were selected on SC-Trp + 2% (w/v) glucose agar plates and cultures were stored at -80°C.

A 10 L starter culture was grown in SC-Trp + 2% (w/v) glucose at 30°C with shaking at 225 rpm overnight and used to inoculate a bioreactor with 100 L of YPG media containing 0.1% (w/v) glucose. Expression of the carrier domain was induced after 24 hours with 0.4% (w/v) galactose and cells were harvested after 4 hours by centrifugation. Cell pellets were washed with MilliQ water and stored at -80°C. Mitochondria were isolated as described above. One kg of yeast cells expressing the carrier domain yielded 4-5 g of mitochondria.

The carrier domain of citrin was purified following a similar procedure as used for full-length citrin with the following modifications. Mitochondria were solubilised with 2% (w/v) DDM (Glycon) and 2.5 mM DTT. All buffers were supplemented with 0.03% (w/v) DDM, 0.03 mg ml^{-1} TOCL and 2.5 mM DTT. On-column digestion was performed with 100 μg Factor Xa in the presence of 20 mM imidazole and 5 mM $CaCl_2$. The mobile phase with untagged carrier domain was separated from the resin using empty midi spin columns at 500g for 5 min. An additional 1.5 ml buffer B was passed through the dry resin to recover residual protein. Purified protein was concentrated using a 100 kDa MWCO centrifugal concentrator at 3,000g. Peak fractions of the size-exclusion step were pooled and concentrated to approximately 9.5 mg ml^{-1} with a 100 kDa MWCO centrifugal concentrator.

Expression and purification of yeast ADP/ATP carrier isoform 2

Aac2p was expressed and purified as previously described with minor modifications[7]. Hepes was used as a buffer component in all buffers. All wash buffers contained 0.03% (w/v) DDM and 0.03 mg ml^{-1} TOCL. The final sample was not concentrated.

Supplementary References

1. Robinson, A.J., Overy, C. & Kunji, E.R. The mechanism of transport by mitochondrial carriers based on analysis of symmetry. *Proc. Natl. Acad. Sci. U. S. A.* 105, 17766-17771 (2008).

2. Afonine, P.V. et al. Towards automated crystallographic structure refinement with phenix.refine. *Acta Crystallogr. D Biol. Crystallogr.* 68, 352-367 (2012).

3. Kyte, J. & Doolittle, R.F. A simple method for displaying the hydropathic character of a protein. *J. Mol. Biol.* 157, 105-132 (1982).

4. Landau, M. et al. ConSurf 2005: the projection of evolutionary conservation scores of residues on protein structures. *Nucleic Acids Res.* 33, W299-302 (2005).

5. Gautier, R., Douguet, D., Antonny, B. & Drin, G. HELIQUEST: a web server to screen sequences with specific alpha-helical properties. *Bioinformatics* 24, 2101-2102 (2008).

6. Gietz, R.D. & Schiestl, R.H. Quick and easy yeast transformation using the LiAc/SS carrier DNA/PEG method. *Nat. Protoc.* 2, 35-37 (2007).

7. Ruprecht, J.J. et al. Structures of yeast mitochondrial ADP/ATP carriers support a domain-based alternating-access transport mechanism. *Proc. Natl. Acad. Sci. U. S. A.* 111, E426-434 (2014).
